# Molecular Representation and Closed-Loop Validation for Toxicity Assessment of Organic Compounds in Ambient Air PM_2.5_

**DOI:** 10.1021/acs.est.5c17667

**Published:** 2026-02-25

**Authors:** Yao Lu, Yangyang Wu, Xiangdong Li

**Affiliations:** a Department of Civil and Environmental Engineering, 26680 The Hong Kong Polytechnic University, Hung Hom, Kowloon,999077 Hong Kong,China; b The Hong Kong Polytechnic University Shenzhen Research Institute, Shenzhen, Guangdong 518057, China

**Keywords:** PM_2.5_, interpretable machine learning, customized molecular fingerprints, closed-loop validation, toxicity assessment, nontargeted analysis, molecular substructures

## Abstract

Although the health impacts of fine particulate matter (PM_2.5_) are primarily attributed to its chemically diverse composition rather than to mass concentration, assessing the toxicity of PM_2.5_ constituent compounds remains highly challenging due to chemical complexity and limited experimental scalability. This study introduces an interpretable machine learning (ML) framework using a curated A549 cytotoxicity data set (19,841 compounds) that integrates customized Molecular Access System (MACCS) fingerprints, six base models, and a stacking ensemble meta-model, all optimized with a biobjective strategy to assess the toxicity of organic compounds in PM_2.5_. The stacking ensemble model demonstrated satisfactory performance (test AUC > 0.8), exhibiting good generalization, adaptability, and robustness. Nontargeted analysis generated and experimentally validated a prediction set from the Hong Kong PM_2.5_ samples (51 of 387 compounds confirmed from 13 classes), demonstrating broad applicability on an independent Nanjing PM_2.5_ set (572 compounds). Key substructures driving toxicity, identified through a SHapley Additive exPlanations (SHAP) analysis and cell experimental validation, revealed that PM_2.5_ compounds with aromatic rings and nitrogen-based functional groups (e.g., aromatic α,β-unsaturated ketones, aromatic amines, aromatic nitro compounds, and tertiary amines) likely contribute to high toxicity. The derived “structure–toxicity” rules narrow the search space from thousands of compounds to those containing critical substructures, enabling efficient prioritization of toxic components and providing a foundation for improving model specificity and predictive accuracy in future studies.

## Graphical abstract

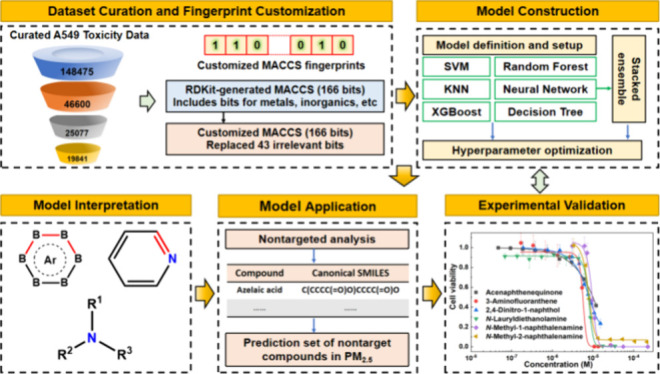

## Introduction

Fine particulate matter (PM_2.5_), one of the major pollutants in the atmospheric environment, has garnered significant attention because of its profound threats to human health.^1,2^ With a diameter of 2.5 μm or less than 2.5 μm, PM_2.5_ can penetrate deep into the lungs and even enter the bloodstream, leading to acute and chronic health problems such as respiratory, cardiovascular, and neurological diseases.^3,4^ Regulatory agencies commonly adhere to the World Health Organization (WHO) guidelines on PM_2.5_ mass concentrations, yet the evidence suggests that spatiotemporal variations in the chemical compositions of PM_2.5_ lead to differing health impacts.^1,5–7^ For instance, in Beijing and Guangzhou during the month of January 2014, toxic metals and polycyclic aromatic hydrocarbons (PAHs) accounted for less than 40% of the oxidative potential of PM_2.5_, underscoring the importance of identifying additional toxic components.^5^ However, PM_2.5_ comprises thousands of chemically diverse compounds, making the identification of its key toxic constituents highly challenging due to the limited availability of comprehensive data and advanced analytical techniques.^8–11^ Currently, robust data and effective control strategies are still lacking, meaning that numerous nontarget compounds remain unidentified and their toxic effects are largely unassessed.^1,3,5^

Traditional toxicity assessment methods predominantly rely on laboratory-based experiments, including *in vitro* toxicity assays and *in vivo* animal studies.^12,13^ These approaches are not only time-consuming, labor-intensive, and costly but also lead to difficulties in comprehensively addressing all potential compounds when evaluating complex pollutant mixtures.^14^ In light of these challenges, the development of efficient, accurate, and interpretable machine learning (ML) methods has become a central focus in contemporary environmental research.^15–20^ For example, ML approaches have been utilized for high-resolution modeling of particulate matter chemical composition and source apportionment.^21–24^ Additionally, ML models have enabled hazard-driven prioritization of features in nontarget screening of environmental water samples,^25^ as well as predictions of PM_2.5_ concentrations in diverse settings, including indoor environments during wildfire smoke events,^26^ urban agglomerations with complex terrain,^27^ and regional scales in the eastern Mediterranean.^28^ Furthermore, a network-based framework combined with ML has been employed to identify key toxic compounds in the airborne chemical exposome linked to cardiovascular diseases.^29^ Quantitative structure–activity relationship (QSAR) approaches have also been widely employed to predict the toxicity of diverse organic chemicals across multiple species—such as algae, daphnia, fish, and bacteria.^30,31^ Furthermore, ML-based nano-quantitative structure–toxicity relationship (nano-QSTR) models have been developed to predict the cytotoxicity of engineered nanomaterials on human lung cells.^32^ Other works have proposed two-stage ML frameworks to predict points of departure (POD), thereby improving the reliability of POD estimation for human noncancer health risk assessment,^33^ as well as ensemble learning-based QSAR models that leverage molecular descriptors to achieve robust and interpretable predictions of human intestinal absorption.^34^ These studies demonstrate the growing application of ML and QSAR in toxicity assessment. However, they primarily focus on general organic chemicals or other environmental matrices, such as aquatic systems, with limited direct relevance to the cytotoxicity prediction of organic compounds in PM_2.5_, and often lack integration with experimental validation for air particulate matter scenarios.

Numerous challenges are still encountered when existing ML models are applied to the toxicity assessment of compounds in PM_2.5_. First, the processing and integration of multisource data to establish an ML data set for PM_2.5_ compound toxicity prediction represents a critical challenge. Currently, systematic approaches for curating data in this domain remain largely underexplored, necessitating the development of effective data integration methods and the construction of relevant databases to create more robust and generalizable predictive models. Second, the effective representation of the structural information on compounds within the data set, including the selection of appropriate molecular fingerprints for modeling to enhance performance, as well as the interpretability of the model, presents another substantial challenge.^35^ The interpretability of the model is critical, especially in the domain of environmental health, where transparency and clarity in decision-making can enhance the credibility and applicability of research findings.^17,36^ Furthermore, a major limitation of most previous studies is their exclusive reliance on open-access data for ML modeling and prediction without systematic and experimental validation. This absence of experimental verification undermines both the predictive accuracy and the real-world applicability of the proposed models.^17,37^

To address the aforementioned challenges, this study presents an interpretable ML model for assessing the toxicity of nontarget organic compounds from authentic PM_2.5_ samples, as illustrated in the overall workflow in Figure 1. The framework integrates customized Molecular Access System (MACCS) fingerprints encoding substructures specific to the curated A549 cytotoxicity data set, a stacking ensemble meta-model that balances prediction robustness with substructure-based interpretability, and a closed-loop validation framework linking computational predictions to experimental toxicity assays. This approach not only enhances interpretability but also achieves superior alignment with experimental data compared with prior database-reliant models. This methodology demonstrates two key capabilities: predicting the toxicity of PM_2.5_ organic compounds and deciphering their toxicity-driving substructures through a SHapley Additive exPlanations­(SHAP) analysis. Unlike conventional models that prioritize performance metrics alone, our biobjective optimization strategy explicitly aligns ML outputs with experimental toxicological evidence—a prerequisite for regulatory credibility and applications. By addressing the “black-box” dilemma in a PM_2.5_ toxicity assessment, this study bridges critical methodological gaps in the transition from mass-dominant to toxicologically significant component-driven PM_2.5_ risk evaluations, thereby laying the mechanistic foundation for the future development of precise source attribution models and context-specific mitigation strategies across global air pollution contexts.

**Figure 1:**
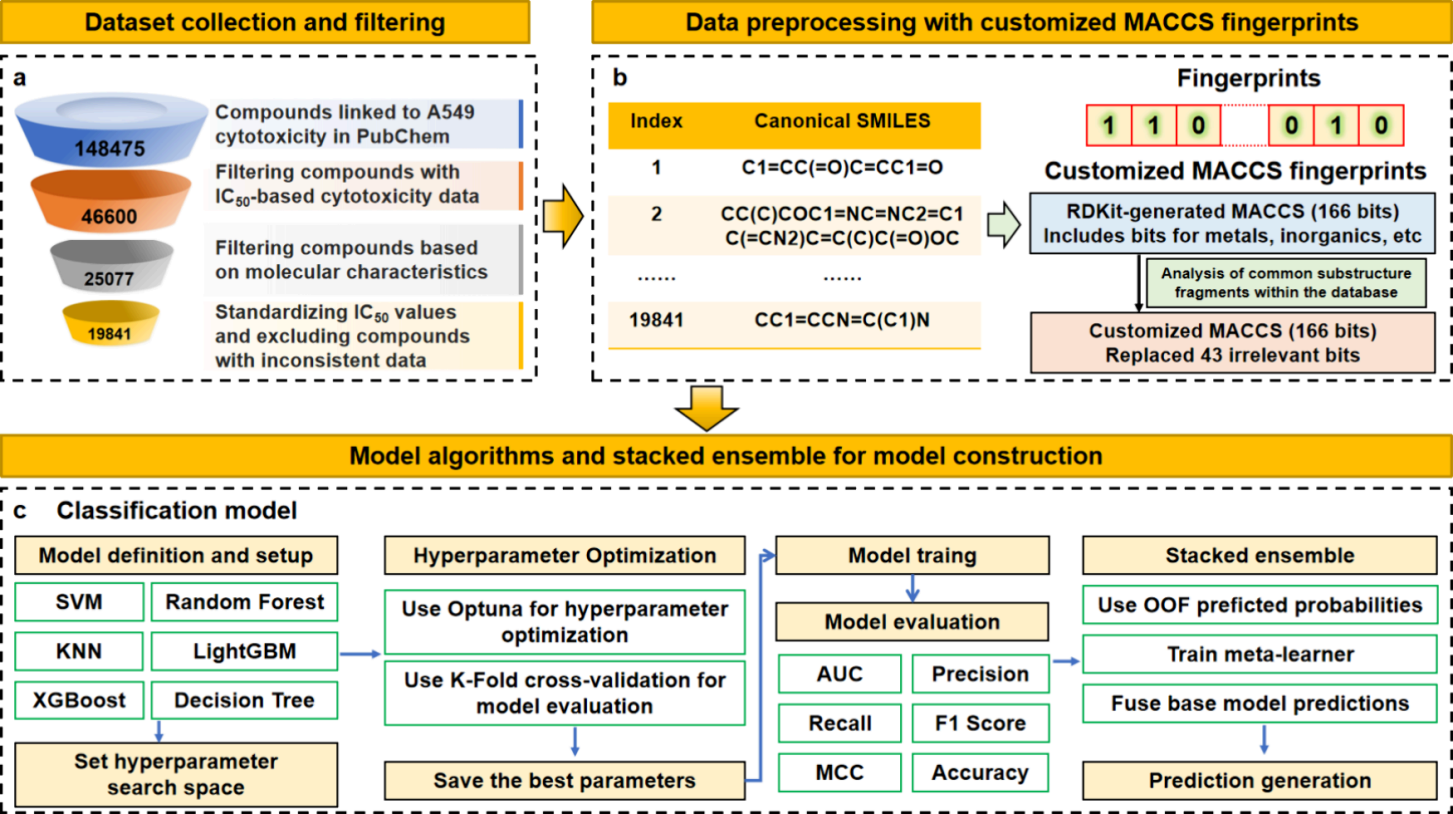
Workflow for predicting the toxicity of compounds in PM_2.5_ using machine learning and experimental validation, including the following key steps: (a) collection and filtering of an A549 cell cytotoxicity database; (b) generation of input features derived from customized MACCS fingerprints; (c) construction of the machine learning model using a stacking ensemble strategy.

## Materials and Methods

### Data Collection and Filtering

Data were primarily sourced from PubChem (https://pubchem.ncbi.nlm.nih.gov/), resulting in 148,475 compounds associated with A549 cytotoxicity. A stringent multistep filtering process was employed, encompassing half-maximal inhibitory concentration (IC_50_) value selection, molecular characteristic filtering, and data standardization. This process refined the data set to 19,841 high-quality organic compounds. Detailed descriptions of the data collection, filtering criteria, and standardization processes are listed in Text S1 of the Supporting Information (SI), with the multistep filtering process illustrated in Figure 1a.

### Data Preprocessing with Customized MACCS Fingerprints

Standard MACCS fingerprints, as defined in the RDKit package (https://www.rdkit.org), consist of 166 bits, with each bit representing a specific molecular substructure or feature. To address the presence of irrelevant inorganic and metal-related bits that lowered the ability to capture PM_2.5_-related organic compounds, as well as the highly scattered bit value distribution that diminished the emphasis on critical features, we developed a customized set of MACCS fingerprints tailored to the A549 data set. Using the canonical Simplified Molecular Input Line Entry System (SMILES) molecular structure information, a series of molecular substructures were identified and incorporated into a custom dictionary. Irrelevant features, such as those related to inorganic and metal-containing compounds, were systematically removed or replaced. The added bits were curated to ensure specificity and precision without redundancy. A total of 43 bits (26% of the original MACCS dictionary) were replaced to enhance the specificity and interpretability. The approach of preprocessing data using customized MACCS fingerprints is illustrated in Figure 1b, with detailed descriptions of feature selection presented in Table S1 of the SI.

### Model Development, Evaluation, and Optimization

Based on an A549 data set comprising 19,841 compounds and the customized MACCS fingerprints described above, a classification ML model was developed to predict the toxicity of nontarget organic compounds in PM_2.5_. This model was fundamentally built upon the quantitative cytotoxicity data (IC_50_) of these known organic compounds, learning the underlying “structure–toxicity” relationships to enable predictions for organic compounds in PM_2.5_. Classification labels were defined by setting a logarithmic IC_50_ (in nM) value of 4 (IC_50_ = 1.0 × 10^4^ nM) as the threshold to distinguish high toxicity (categorized as 1) from low toxicity (categorized as 0). This threshold was selected because compounds with IC_50_ values of below 1.0 × 10^4^ nM are generally regarded as possessing marked cytotoxic potential in cell-based assays, a well-established standard in multiple toxicological studies.^38,39^ Furthermore, an analysis of the IC_50_ distribution within our A549 cell toxicity data set confirmed that a threshold of logarithmic IC_50_ value of 4 effectively differentiates compounds with varying toxicity levels (Figure S1 of the SI), thereby validating the appropriateness of this threshold.

The following six ML algorithms were used as base models: Support Vector Machine (SVM), Random Forest, K-Nearest Neighbors (KNN), LightGBM, XGBoost, and Decision Tree classifiers. The data set was randomly split into training (90%) and testing (10%) sets. This ratio was chosen to maximize the data available for model training and hyperparameter tuning while reserving a holdout set for unbiased final evaluation. In the training portion, 5-fold cross-validation was performed to capture patterns more reliably and reduce variance in performance estimates, thereby supporting robust model construction. Hyperparameter optimization of the model was performed through an automated search using an Optuna to train and evaluate the model. This process was initially performed 300 times for each of the six base models, with the best hyperparameters selected and fixed. Proper hyperparameter tuning prevents the model from being overly simplistic, which could lead to underfitting and poor generalization, or excessively complex, which could result in overfitting through the capturing of noise in the training data. Identifying appropriate hyperparameters can enhance the model’s performance metrics, such as accuracy and recall.

Stacking ensemble learning was employed in the present study. The stacking algorithm initially trained multiple base models and utilized their predictions to generate new features. These features were then integrated by a meta-model to form a more robust ensemble model. Due to its computational efficiency, robustness, and interpretability, logistic regression was selected as the meta-model learning algorithm for the stacking ensemble.^40,41^ Furthermore, hyperparameter optimization of the logistic regression model was conducted using Optuna, to achieve an optimal alignment between the performance metrics of the stacking ensemble and the external validation results.^42^ After tuning of 70 sets of hyperparameters, the optimal configuration for the logistic regression algorithm was identified.

The performance evaluation of the six algorithm models, as well as the stacking ensemble model, was carried out using 5-fold cross-validation on the 90% training set, where it was divided into five equal subsets; the model was trained on four subsets and validated on the fifth, repeating this five times. The 10% test set, entirely excluded from training and validation, was used solely for final model evaluation to assess generalization. Model performance was assessed using metrics such as Area Under the Curve (AUC), Precision, Recall, F1 Score, Matthews Correlation Coefficient (MCC), and Accuracy.^43^ Prediction uncertainty was further quantified using binary entropy of the predicted probabilities, as detailed in Text S2 of the SI. In the prediction phase, the trained stacking model was applied to an external new data set to perform classification predictions, outputting predicted labels, and corresponding probabilities. The results were then saved to an output file for subsequent analysis. It is important to note that there are many situations in ML that involve random elements, such as data splitting, model initialization, data augmentation, hyperparameter searches, and various aspects of ensemble learning. Without setting a random seed, the model training process will yield different results each time, meaning that the program lacks reproducibility. Therefore, it is essential to set a random seed in the program to ensure the reproducibility of experiments and prevent fluctuations in model performance due to random variations. The workflow for the model algorithms and the stacking ensemble for the development, evaluation, and optimization of the model is illustrated in Figure 1c.

### Model Interpretation

To improve the interpretability of the model with respect to its chemical features, this study integrated the SHAP method with clustering techniques.^44^ This approach involved analyzing the importance of both overall and specific features, identifying key molecular substructures relevant to toxicity predictions and visually representing the relationships between features and prediction outcomes. Details of the methodology for a SHAP analysis combined with clustering techniques are provided in Text S3 of the SI.

### PM_2.5_ Sample Collection and Nontargeted Organic Compound Analysis

PM_2.5_ samples were collected from five sampling sites in Hong Kong in 2022 and 2023, namely, an ambient site and a roadside site at The Hong Kong Polytechnic University (PolyU), as well as the Kwai Chung, Tung Chung, and Southern District Environmental Protection Department (EPD) Sampling Stations. Additionally, PM_2.5_ samples from the urban Xuanwu and rural Lishui sites in Nanjing, China, covering 2019 to 2021, were included. Detailed information on the sampling sites, methods, dates, and PM_2.5_ concentrations is presented in Texts S4 and S5, as well as in Table S2 of SI.

PM_2.5_ samples were pretreated using established protocols^45–47^ with some modifications and subjected to ultrahigh performance liquid chromatography coupled with Orbitrap mass spectrometry (UHPLC-Orbitrap MS), as described in Text S6 of the SI. The resulting data were processed by using Compound Discoverer software. Databases such as ChemSpider and MzCloud were employed to identify molecular structures and fragmentation patterns, with details provided in Text S7 of the SI. Additionally, the *in silico* fragmentation tool MetFrag was employed as a supplementary tool for identifying nontarget organic compounds,^48^ with details provided in Text S8 of the SI. The compounds that were identified were assigned confidence levels (CL) ranging from 1 to 5 based on predefined criteria,^49^ with CL1 compounds confirmed using tandem mass spectrometry (MS^2^). Retention times and characteristic fragmentation patterns were compared with those of the standard compounds. By integrating these tools and databases, a prediction set of organic compounds in PM_2.5_, encompassing CL1, CL2, and CL3, was generated.

### Validation of Toxicity Predictions Using *In Vitro* Assays

To validate the toxicity predictions generated by ML models, *in vitro* assays were performed with human lung epithelial cell line A549 obtained from the American Type Culture Collection (ATCC). Details of the tested compounds classified as CL1 through a nontargeted analysis are provided in Table S3 of the SI. Cell culture experiments included seeding A549 cells in 96-well plates and assessing cell viability with 3-(4,5-dimethylthiazol-2-yl)-5-(3-carboxymethoxyphenyl)-2-(4-sulfophenyl)-2*H*-tetrazolium (MTS) assays to determine the IC_50_ values of the tested compounds.^50,51^ Comprehensive information on cell culture conditions and experimental protocols is available in Text S9 of the SI.

## Results and Discussion

### Data Set Curation and Fingerprint Customization

Constructing a high-precision ML data set for predicting PM_2.5_ toxicity by extracting toxicity data from diverse sources presents a substantial challenge, as the relevance and suitability of the data for PM_2.5_ research are crucial, with both the quantity and quality of the data set being essential for enhancing the robustness and predictive accuracy of the ML model.^17,52^ To address this issue, we adopted an integrated approach that combined public database surveys and literature mining to select A549 cell viability data, which are both abundant and widely used in PM_2.5_ toxicity studies. The A549 cell line, a human alveolar epithelial carcinoma model with documented sensitivity to airborne pollutants, served as a relevant *in vitro* system for assessing PM_2.5_-induced respiratory toxicity due to its relevance to inhalation exposure mechanisms.^50,53^ Through multistage quality control measures (Figure 1a and Text S1 of the SI), including IC_50_ data filtering, PM_2.5_ molecular characteristic-based filtering, and data standardization, we ultimately constructed a high-quality data set consisting of 19,841 organic compounds associated with A549 cell toxicity.

To enable effective ML modeling on the constructed high-quality database, molecular fingerprints are essential as a key feature representation method.^35^ We initially selected the MACCS fingerprint due to its clear feature definitions and high interpretability, making it particularly suitable for identifying molecular substructures of organic compounds related to cell toxicity.^54^ However, the standard MACCS fingerprint exhibited issues of feature redundancy and insufficient correlation when applied to our A549 cell toxicity data set. To address this issue, we customized the MACCS fingerprints by removing inorganic and metal-related fragments irrelevant to organic molecular toxicity and incorporating additional substructures specifically associated with organic toxicity (Figure 1b and Table S2 of the SI). This customization process not only enhanced the relevance and specificity of the features but also improved the interpretability of the fingerprints, enabling the ML models to identify more relevant key toxicological features.

To thoroughly evaluate the A549 data set, we first analyzed the toxicity distribution. This analysis revealed that the IC_50_ values of the compounds exhibited a broad range of toxicity levels from low to high, with $\log_{10}\left(\frac{IC_{50}}{\mathrm{nM}}\right)$ values ranging between −3 and 8. The majority of compounds were concentrated in the $\log_{10}\left(\frac{IC_{50}}{\mathrm{nM}}\right)$ range of 3 to 5, comprising 11,781 low-toxicity compounds ( $\log_{10}\left(\frac{IC_{50}}{\mathrm{nM}}\right)$ ≥ 4, categorized as 0), accounting for 59.4% of the total compounds, and 8,060 high-toxicity compounds ( $\log_{10}\left(\frac{IC_{50}}{\mathrm{nM}}\right)$ < 4, categorized as 1), constituting 40.6% ( of the ). This distribution reflected a balanced and comprehensive toxicity profile, supporting the data set’s reliability and suitability for constructing toxicity classification models. Additionally, we evaluated the structural diversity of the data set by performing a Tanimoto similarity analysis on the 19,841 compounds represented by customized MACCS fingerprints ( of the ). The results indicated high structural diversity within the A549 database, demonstrated by the clustering of compound similarities predominantly between 0.3 and 0.5, with relatively few pairs exceeding a similarity of 0.6. The peak at a similarity of 0.4 suggested that the compound selection process effectively reduced redundancy, thereby enhancing the chemical space coverage and maintaining high structural diversity. This combination of balanced toxicity distribution and robust chemical diversity facilitates the development of robust and widely applicable ML models for toxicity prediction in PM_2.5_ studies.

### Machine Learning Model Development and Performance

Building on the A549 cell toxicity data set and customized MACCS fingerprints, we developed ML classification models to predict the toxicity of compounds in PM_2.5_, as illustrated in Figure 1c, which outlines the model development, evaluation, and optimization process. The model was trained on a randomly selected subset of 17,857 compounds (90% of the total). The high structural diversity of the entire data set ensured that the training set was representative of a broad chemical space (Figure S2 of the SI), which guaranteed that the learned structure–toxicity relationships were robust and generalizable to the diverse organic compounds found in PM_2.5_. A stacking ensemble framework was employed to integrate six base classifiers (SVM, Random Forest, KNN, LightGBM, XGBoost, and Decision Tree), utilizing their predicted class probabilities as input features for a logistic regression-based meta-model, which generated the final toxicity predictions.^40,41,55^ The applicability domain of the model was defined by organic compounds containing carbon (C), hydrogen (H), oxygen (O), nitrogen (N), and sulfur (S), with molecular weights ranging from 50 to 900 Da. The structural constraints included H/C ratios from 0.3 to 3.0, O/C ratios from 0.0 to 3.0, N/C ratios from 0.0 to 1.3, S/C ratios from 0.0 to 0.8, and double bond equivalent (DBE)/C ratios from 0.0 to 1.0, consistent with typical PM_2.5_ organics detected through nontargeted chemical analysis.^45–47^

Hyperparameter optimization was conducted using Optuna,^42^ with a biobjective strategy aimed at maximizing both base and meta-model performance and alignment with experimental toxicity data. Key metrics—AUC, precision, recall, F1 score, and accuracy—were optimized, with the training set exceeding an AUC of 0.9, and validation/test sets surpassing 0.8, while minimizing the gap between them. This optimization utilized *in vitro* toxicity data from 51 compounds identified through the nontargeted analysis of PM_2.5_, categorized into 13 chemical classes (Figures 4c and 5a,b and Table S8 of the SI), which are described in detail in subsequent sections.

Differences were observed between the performances of the six base models and the stacking ensemble model on the A549 cell toxicity data set (Figure 2a,b, Table S4 of the SI). Specifically, SVM, Random Forest, LightGBM, and XGBoost exhibited strong performance on the test set, as indicated by their high F1 scores, while the KNN algorithm underperformed relative to the aforementioned models. The Decision Tree exhibited the weakest performance, which might be attributed to its simpler architecture, as evidenced by its relatively lower F1 score (Figure 2a). Although the stacking ensemble model demonstrated a slightly lower test set performance compared with the individual models (Random Forest, LightGBM, and XGBoost; Figure 2a,b), its integration of diverse base learners substantially enhanced its robustness against shifts in data distribution. By strategically combining the strengths of individual models and mitigating single-model biases, the stacking framework offers a more reliable solution for real-world toxicity prediction scenarios, where data heterogeneity is unavoidable. Confusion matrices (Figures S3 of the SI and Figure 2c) confirmed good generalizability, with 94.9% training accuracy (16,947/17,857) and 78.1% test accuracy (1,551/1,985), underscoring the robust performance of the stacking ensemble model, particularly in class-imbalanced scenarios. Furthermore, to validate the advantages of our customized MACCS fingerprints, we performed a systematic comparison against standard MACCS fingerprints and RDKit 2D molecular descriptors using the database of 19,841 compounds. The performance of ML models across these feature sets (Tables S4, S5, and S6 of the SI) demonstrated the clear superiority of our approach. This was evidenced by consistent and marked improvements in internal validation when compared to standard MACCS fingerprints. More critically, the customized MACCS fingerprints exhibited substantially enhanced generalization capability on the external experimental set of PM_2.5_-identified compounds evaluated by A549 cytotoxicity assays, as described below, significantly outperforming the molecular descriptors, which showed notably limited applicability. The robustness of this conclusion was further underscored by the stacking ensemble model, which maintained strong predictive performance with our customized MACCS fingerprints on the experimental set but experienced a complete loss of efficacy when relying on the conventional RDKit 2D descriptors. These results collectively confirm that our structural modifications are indispensable for achieving high and generalizable performance in predicting the PM_2.5_ toxicity of organic compounds.

**Figure 2:**
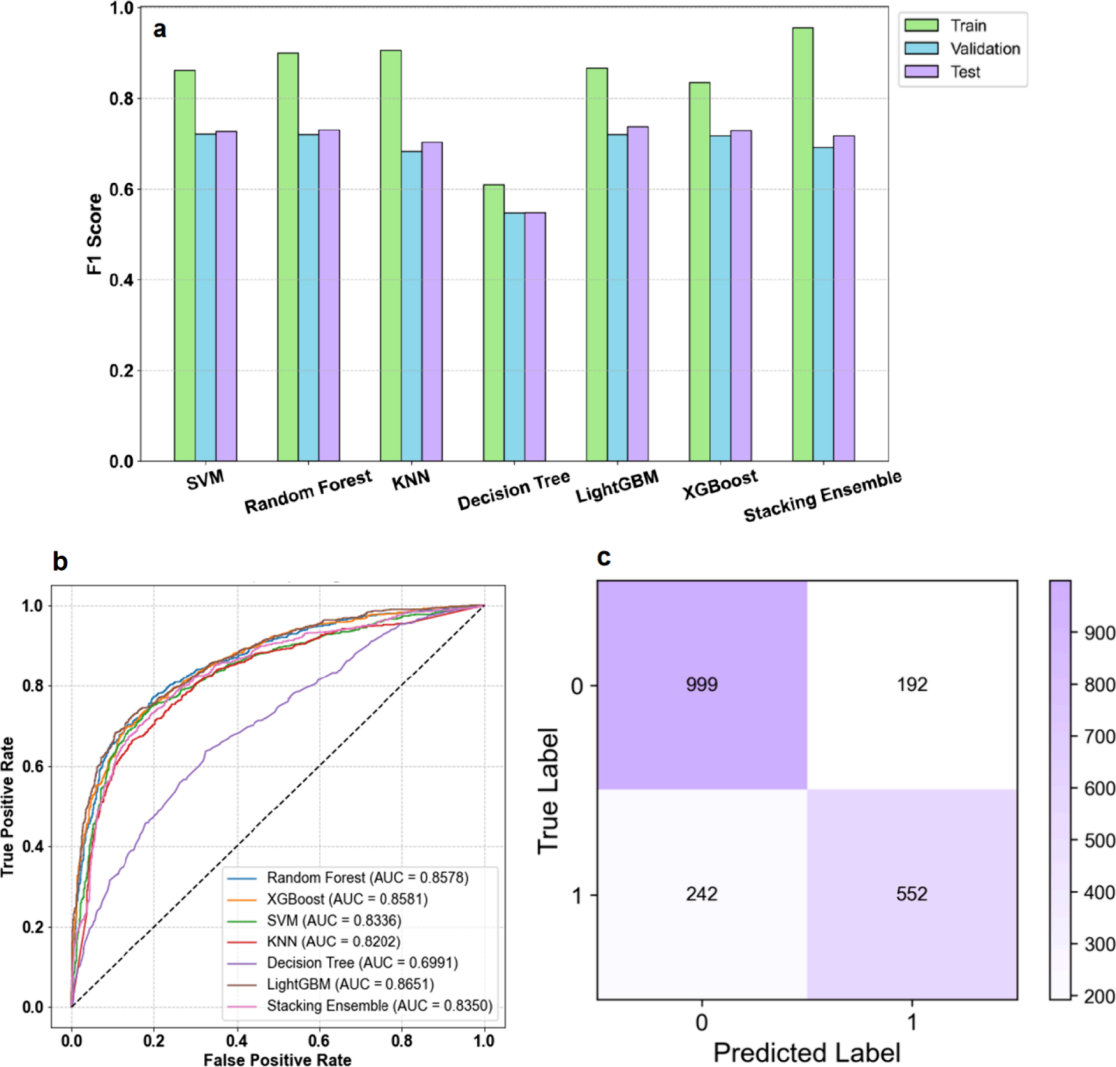
Performance evaluation of machine learning (ML) algorithms on the A549 cell toxicity data set: (a) F1 scores across training, validation, and test data sets; (b) receiver operating characteristic (ROC) curves and area under the curve (AUC) scores on the test data set for SVM, Random Forest, KNN, Decision Tree, Light Gradient Boosting Machine (LightGBM), Extreme Gradient Boosting (XGBoost), and the stacking ensemble model; (c) confusion matrix of the stacking ensemble model on the test data set.

### Model Interpretation

One major limitation of current ML research is the lack of scientific interpretability of its prediction results, which prevents the results from being applied to regulatory applications in air pollution control and management.^42^ In toxicology research, the development of models that provide reliable explanations is prioritized over the mere optimization of performance.^37^ To address this issue, we employed the SHAP method to interpret the outcomes of the stacking ensemble model.^44^ SHAP facilitated the visualization of key molecular features that influenced toxicity predictions, providing qualitative insights and quantitative evaluations of the critical substructures. This analysis directly revealed the specific structural features that the model identified in the training data as indicators of high toxicity, thereby bridging the gap between the model’s predictions and known toxicological substructures.

Figure 3a presents the top 20 representative molecular features identified by the stacking ensemble model and their corresponding SHAP values, with positive SHAP values indicating a higher probability of high toxicity and negative SHAP values indicating a higher probability of low toxicity. Notably, among these top 20 molecular features, the blue-highlighted “bit 6”, “bit 46”, “bit 103”, “bit 129”, and “bit 165” in Figure 3b were derived from our customized MACCS fingerprints. This finding highlights the contribution of our customized MACCS fingerprints to improving the interpretability of the model.

**Figure 3:**
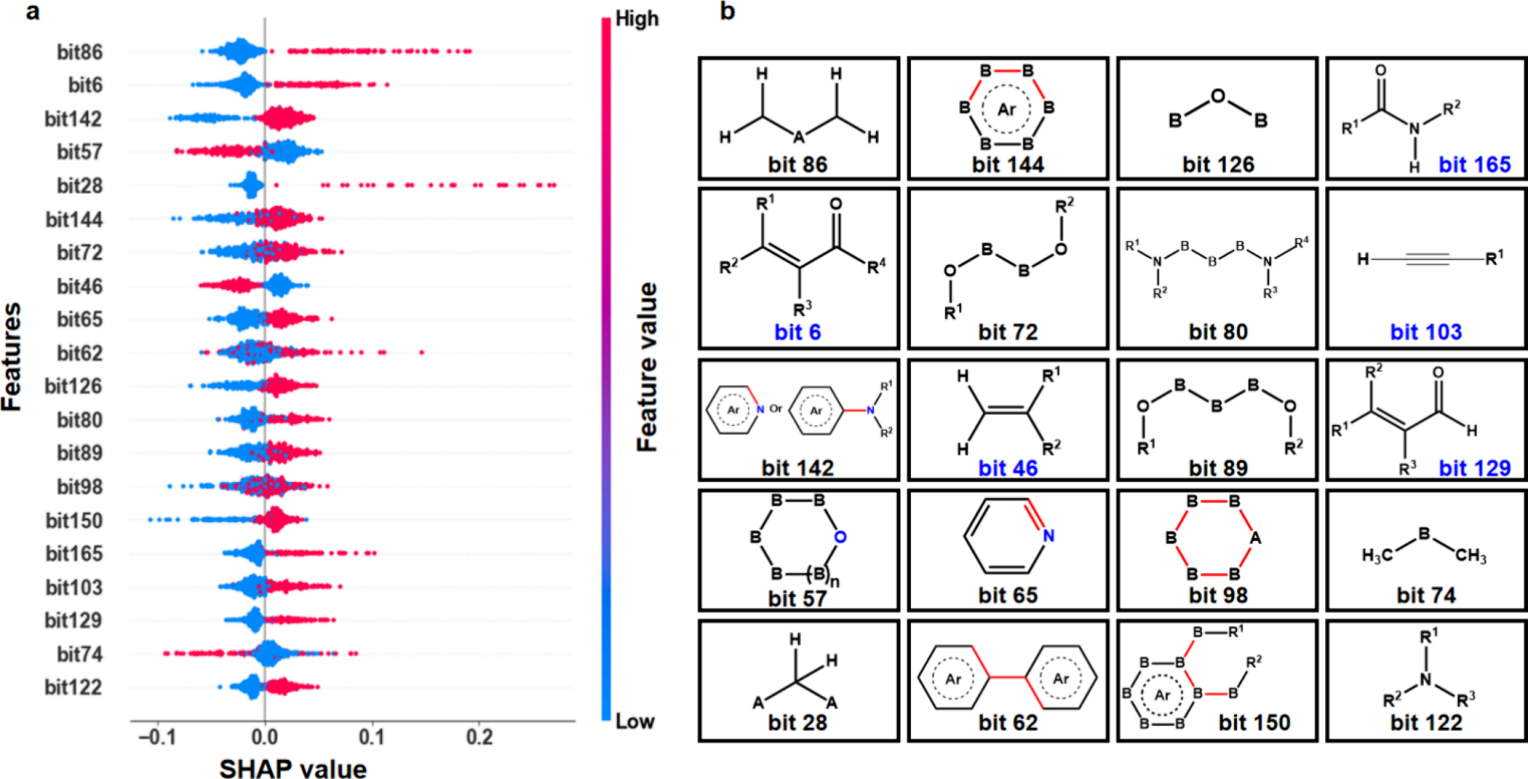
Representative fingerprints and their corresponding chemical substructures in the stacking ensemble model: (a) The top 20 representative fingerprints and their corresponding SHAP values in the stacking ensemble model. (b) Chemical substructures represented by the 20 representative fingerprints, where A represents noncarbon, non-hydrogen atoms; B represents any atom; N represents nitrogen atoms; O represents oxygen atoms; R represents any substituent group, with the number in the upper right of R indicating the label of the substituent group; Ar represents aromatic structures; and red bonds indicate that the fingerprint-bit fragment only includes the red portion, while the black structural fragments are included to highlight the meaning of the red bonds.

A further analysis of the molecular substructures in Figure 3b revealed several commonalities and characteristics among these sites: (1) The sites bit 142, bit 144, bit 65, bit 62, and bit 150 all displayed characteristics consistent with an aromatic ring (Ar) structure. (2) The features of bit 142 and bit 65 included nitrogen (N) atoms associated with the Ar structure; furthermore, bit 80, bit 165, and bit 122 also exhibited N atom characteristics, corresponding to amines or amides. (3) Bit 6 and bit 129 demonstrated characteristics of α,β-unsaturated ketones. (4) Bit 57 and bit 98 showed characteristics of heterocyclic structures, while bit 142 and bit 65, containing nitrogen (N) in Ar structures, also belonged to heterocycles. (5) Bit 86 and bit 103 demonstrated characteristics of alkyl substituents or triple bond structures. These findings suggest that aromatic ring structures may play a crucial role in molecular features related to cell toxicity, implying that nitrogen-containing groups associated with aromatic structures may play a pivotal role in toxicity. The presence of amines, amides, α,β-unsaturated ketones, and heterocyclic structures may influence toxicity, whereas alkyl groups or highly unsaturated hydrocarbon groups could also contribute to toxicity to some extent. It is worth noting that while SHAP values provided interpretability for toxicity predictions through “bit” features, the representativeness of these “bit” features, as well as the complexity of their molecular structures, may cause the model to overlook or misjudge certain structural characteristics, particularly when handling more complex chemical structures. Nevertheless, this dual representation of SHAP values and molecular substructures has indeed enhanced the transparency of the stacking ensemble model and strengthened its potential for applications in the assessment and management of air PM_2.5_ toxicity.

**Figure 4:**
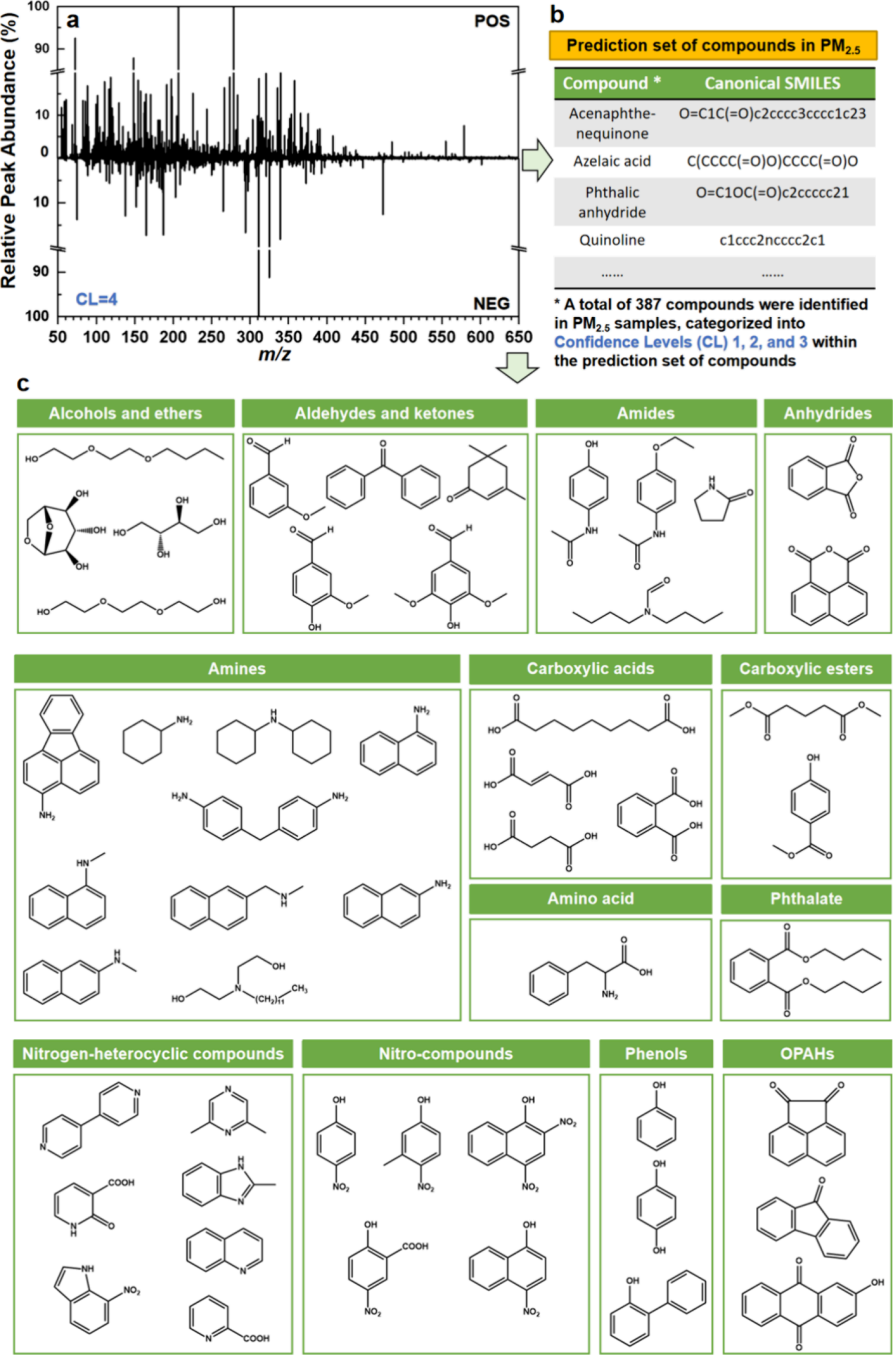
Model application: Predicting the toxicity of nontarget PM_2.5_ compounds using the established model. (a) A high-resolution mass spectrometry (HRMS) reconstruction demonstrates the detection of compounds in Hong Kong PM_2.5_ samples using both positive (ESI^+^) and negative (ESI^–^) electrospray ionization modes, with all detected compounds categorized under Confidence Level (CL) 4. (b) A prediction set was generated from Hong Kong PM_2.5_ samples, with compounds classified into CL1, CL2, and CL3. (c) Summary of validated CL1 compounds confirmed with reference standards, comprising 51 compounds spanning 13 chemical classes.

**Figure 5:**
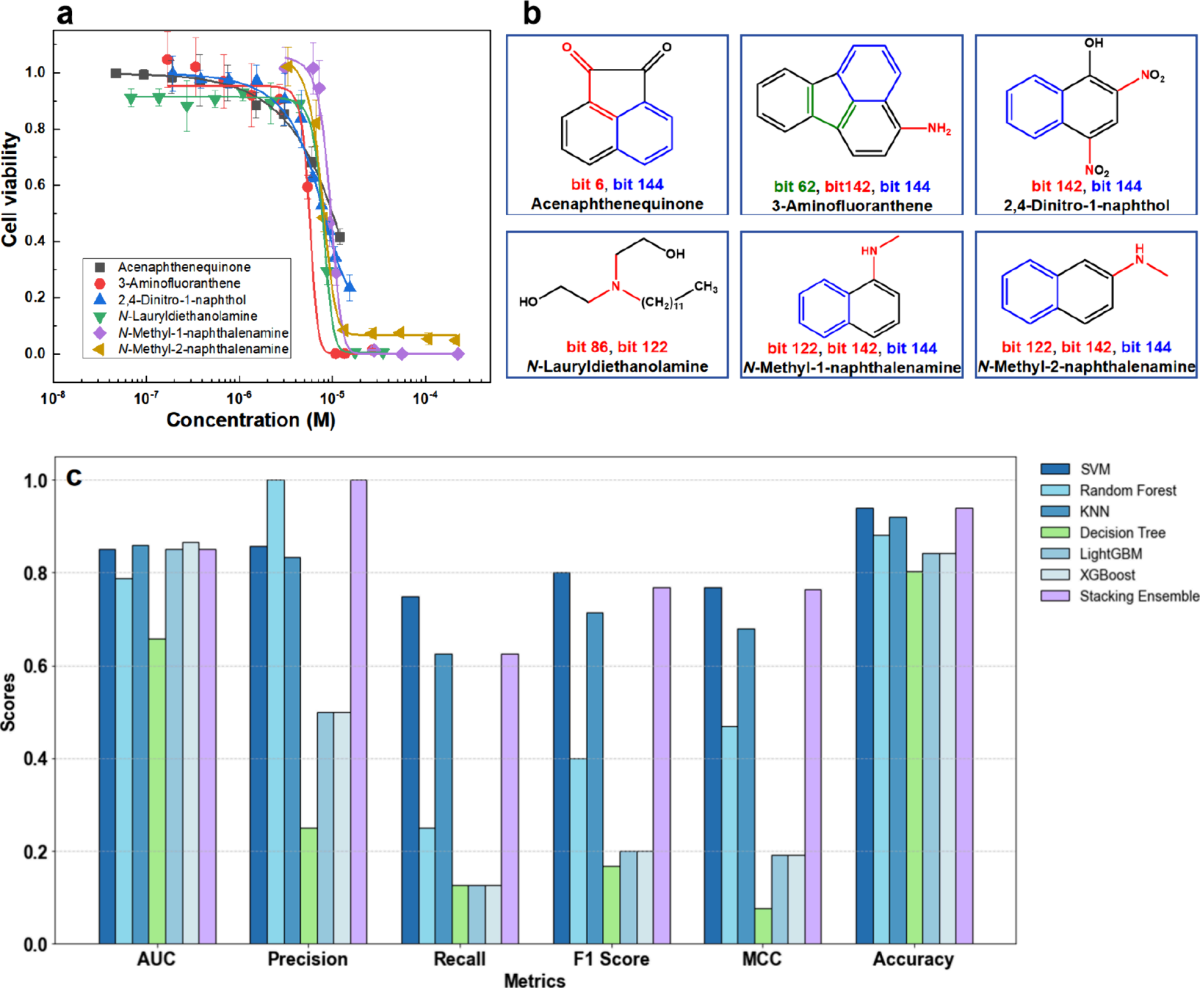
Linking predicted cytotoxicity to molecular substructures through experimental validation. (a) Concentration–effect curves of six compounds categorized by high toxicity (1): OPAH (acenaphthenequinone), amines (3-aminofluoranthene, *N*-lauryldiethanolamine, *N*-methyl-1-naphthalenamine, and *N*-methyl-2-naphthalenamine), and nitro-compound (2,4-dinitro-1-naphthol) on A549 cell viability. (b) Chemical structures of the six compounds with marked fingerprint bits from the top 20 important features based on SHAP values; (c) performance evaluation of ML models on the experimental data sets.

To the best of our knowledge, this study represents an innovative and systematic framework that effectively bridges the gap between predictive performance and mechanistic transparency through the integration of a biobjectively optimized stacking ensemble framework, customized MACCS fingerprints, and SHAP-based interpretability. Compared to conventional QSAR models based on linear descriptors or earlier ML approaches utilizing generic molecular descriptors,^30^ such as the nano-QSTR model that employs tree-based algorithms and physicochemical nanodescriptors for A549 cytotoxicity prediction in nanomaterials,^32^ our method explicitly associates toxicity predictions with the presence or absence of key molecular substructures, as rigorously validated through closed-loop experimental confirmation. This paradigm establishes “structure–toxicity” decision rules that are both machine-actionable and chemically intuitive—representing a significant advancement for screening complex environmental mixtures.

### Nontargeted Chemical Analysis of PM_2.5_ for a Generating Prediction Set for Model Application and Validation

To apply the established ML models for analyzing authentic PM_2.5_ compounds in real-world scenarios, nontargeted analyses were initially performed to generate a prediction set from the detected compounds in Hong Kong PM_2.5_.^10,45–47^ Figure 4a displays the reconstruction mass spectrum obtained from the nontargeted analysis using high-resolution mass spectrometry (HRMS), where each line represents a distinct compound. This reconstruction illustrates unequivocal molecular formulas for the thousands of compounds detected in all of the Hong Kong PM_2.5_ samples included in this study, as indicated by the molecular formulas assigned to CL 4. The further utilization of built-in databases within the Compound Discoverer software, such as ChemSpider and MzCloud, alongside the *in silico* fragmentation tool MetFrag, enabled the elucidation of structures represented by CL4 unequivocal molecular formulas, advancing to CL3 (Tentative Candidates) and CL2 (Probable Structures). These levels, in conjunction with compounds assigned as CL1 through standard verification, collectively formed a prediction set comprising all 387 detected compounds in Hong Kong PM_2.5_, as illustrated in Figure 4b. To achieve CL1 in compound identification, compounds selected from CL2 and CL3 with available commercial standards were validated by comparing retention times, parent ion masses (*m*/*z*), and fragment ion patterns, as detailed in Table S7 of the SI. A total of 51 compounds were confirmed as CL1, encompassing 13 distinct chemical classes, such as alcohols and ethers, aldehydes and ketones, amides, anhydrides, amines, carboxylic acids, amino acids, carboxylic esters, phthalates, nitrogen-heterocyclic compounds, nitro compounds, phenols, and oxygenated polycyclic aromatic hydrocarbons (OPAHs). Figure 4c summarizes these validated CL1 compounds, highlighting the chemical diversity present in PM_2.5_ samples and demonstrating the robustness of the nontargeted analysis approach. The 51 CL1 compounds cover a wide spectrum of functional groups, including aromatic and heterocyclic rings (e.g., acenaphthenequinone and quinoline), nitrogen-containing moieties (e.g., dicyclohexylamine, 5-nitrosalicylic acid, and 2-pyrrolidinone), oxygen-based groups (e.g., 3-methoxybenzaldehyde, 2-phenylphenol, fumaric acid, methylparaben, triethylene glycol, and 1,8-naphthalic anhydride), and multifunctional molecules (e.g., *L*-phenylalanine), thereby offering strong structural representativeness for typical organic constituents of PM_2.5_. Moreover, these compounds align with major chemical classes consistently reported in ambient air PM_2.5_ from various regions—such as phthalates, phenols, and carboxylic esters in Beijing,^56^ nitroaromatics and oxygenated aromatics in central Europe,^57^ and oxygenated organics, nitroaromatics, and carboxylic acids identified in broader multiregional studies^58^—providing a robust real-world basis for model validation. Given the compositional complexity of PM_2.5_ and current sampling constraints, the present validation across 13 compound classes may not capture the full spectrum of airborne organics; to fully enhance the model’s adaptability, future testing should include newly identified compound classes from other regions. Nevertheless, these nontargeted analyses collectively represent a substantial portion of the organic constituents in PM_2.5_, effectively capturing the broad spectrum of organic molecules found in air particulate matter.

### Experimental Validation of Machine Learning Toxicity Predictions

To validate the accuracy of the toxicity predictions made by the ML models, 51 compounds identified as CL1 in the nontargeted analysis, representing 13 distinct chemical classes, were chosen for *in vitro* toxicity assays using the A549 cell line. Critically, the environmental relevance of these compounds is well-established. While some are primary emissions, many are recognized secondary products or semivolatile species that readily partition to the particle phase under realistic atmospheric conditions, consistent with their detection in ambient PM_2.5_ from various regions.^56–58^ Figure 5a presents the concentration-effect curves of six highly toxic (categorized as 1) compounds, specifically including OPAH (acenaphthenequinone), amines (3-aminofluoranthene, *N*-methyl-1-naphthalenamine, *N*-methyl-2-naphthalenamine, and *N*-lauryldiethanolamine), and nitro-compound (2,4-dinitro-1-naphthol), with additional toxicity data provided in Table S8 of the SI. A structural analysis of these six highly toxic compounds revealed that their structures contained bits from the top 20 representative fingerprints identified through the SHAP analysis, as illustrated in Figure 5b. A further analysis of the substructures corresponding to the bits in Figure 5b revealed the presence of aromatic ring structures and nitrogen-containing structures. Specifically, molecular features such as aromatic α,β-unsaturated ketones, aromatic amines, aromatic nitro compounds, and tertiary amines were observed and likely contributed to the high toxicity.

Regarding the matching results of the *in vitro* toxicity experiments and ML predictions for the 51 compounds, as shown in Table S9 of the SI, the stacking ensemble and SVM models both achieved a matching count of 48, demonstrating the highest level of matching accuracy and reliability. The matching count of the Random Forest model was 45, while those of XGBoost and LightGBM were 43, indicating satisfactory results. The matching count of the Decision Tree model was the lowest, at only 41, indicating a relatively poor overall performance.

In the performance evaluation of the ML models on the experimental set, as shown in Figure 5c and detailed in Tables S9 and S10 of the SI, the AUC results demonstrated that the XGBoost, KNN, and SVM models exhibited a strong ability to distinguish between positive and negative samples, whereas the Decision Tree model had a lower AUC, indicating poor suitability for this task. An analysis of Precision and Recall showed that the Random Forest and stacking ensemble models had extremely high prediction accuracy. However, their Recall was relatively low, indicating that although these models could make precise predictions, they had insufficient coverage of positive samples. Comprehensive metrics such as the F1 score and accuracy further showed that the stacking ensemble and SVM models perform best in balancing Precision and Recall. The Decision Tree model, however, had the lowest F1 score, making it the worst-performing model. The MCC metric further validated these findings, with stacking ensemble and SVM achieving significantly better MCC values than the other models, while the Decision Tree model showed the worst MCC performance. A comprehensive analysis demonstrated that both the stacking ensemble and SVM were exemplary models. In practical applications, it is recommended that the stacking ensemble model be prioritized because of its additional advantages, which include superior generalization capabilities and enhanced stability. Incorporating the prediction results of SVM is also recommended, as doing so would allow the strengths of the SVM to be leveraged, and this integrated approach would further enhance overall prediction performance and lead to greater model efficacy.

A further analysis of the prediction results from our stacking ensemble model revealed that, of the 387 identified compounds in the Hong Kong PM_2.5_ prediction set, 25 were predicted to have high toxicity (categorized as 1), accounting for 6.5% of the total, whereas 362 compounds were predicted to have low toxicity (categorized as 0), comprising 93.5% of the total (Figure S4 of the SI). It is worth noting that this observed imbalance in toxicity predictions did not reflect a limitation of the model but rather the specific characteristics of the PM_2.5_ samples analyzed in this study—including their chemical composition and the nontargeted screening methodology—representative of real-world PM_2.5_ samples such as those collected in Hong Kong. Quantitative uncertainty assessment confirmed the high confidence of these predictions, showing that the stacking ensemble achieved the narrowest, tallest-peaked, and most pronounced left-shifted uncertainty distribution (Figure S5 of the SI), delivering the highest-confidence (entropy <0.2, probability <0.09 or >0.91), very high-confidence (entropy <0.3, probability <0.14 or >0.86), and confident (entropy <0.5, probability <0.21 or >0.79) predictions for ∼20.7, ∼36.4, and ∼57.6% of compounds, respectively.^59^ This finding aligned with the understanding that while secondary aerosols dominate PM_2.5_ mass, targeted primary and semivolatile species could exert disproportionate toxic effects due to their high intrinsic potency.^60^ This distribution highlighted the potential utility of the model in identifying a small subset of highly toxic compounds within complex environmental mixtures, thereby enabling a more efficient prioritization and targeted investigation of key toxic components in PM_2.5_. The chemical structures of compounds in PM_2.5_ that were predicted to have high toxicity by our stacking ensemble model, along with the fingerprint bits corresponding to the top 20 representative features identified based on SHAP values, are shown in Figure S6 of the SI. Based on predictions from the ML models, supported by a SHAP analysis and *in vitro* assays, it was concluded that the compounds present in the Hong Kong PM_2.5_ samples, containing aromatic rings and nitrogen-based functional groups—such as aromatic α,β-unsaturated ketones (e.g., acenaphthenequinone), aromatic amines (e.g., *N*-methyl-1-naphthalenamine), aromatic nitro compounds (e.g., 2,4-dinitro-1-naphthol), and tertiary amines (e.g., *N*-lauryldiethanolamine)—were strongly associated with high toxicity. Thus, our results provided a substructure-based perspective that complements the mass-dominant view of PM_2.5_ toxicity, identifying specific high-potency compounds that may originate from both primary and secondary sources.

To demonstrate our ML model’s generalizability for predicting PM_2.5_ toxicity across diverse real-world environments, we tested it on an independent set of PM_2.5_ samples collected from Nanjing, China. This data set covered urban and rural sites, whereas the Hong Kong data set covered a range of distinct urban settings, including traffic, industrial, and coastal environments. Nontargeted analysis of the Nanjing PM_2.5_ samples generated a distinct prediction set of 572 organic compounds. The model identified 92 high-toxicity compounds (16.1%) in this external data set—a proportion notably higher than that in the Hong Kong samples (6.5%)—which likely reflected differences in nontargeted analytical methodologies and distinct source profiles between the two cities. Furthermore, among the 92 compounds predicted as highly toxic in the Nanjing PM_2.5_ samples, the vast majority contained key SHAP-identified toxicity-driving substructures—notably aromatic amines (e.g., *p*-dimethylaminobenzaldehyde), aromatic nitro compounds (e.g., 2,4-dinitrophenol), tertiary amines (e.g., tributylamine), and aromatic α,β-unsaturated ketones (e.g., Michler’s ketone). These substructures closely mirrored those previously confirmed to drive high cytotoxicity in the Hong Kong PM_2.5_ samples, providing direct evidence that the model had learned transferable structure–toxicity rules. The same quantitative uncertainty assessment demonstrated high predictive confidence that was robustly reproduced on this completely independent data set. The stacking ensemble again exhibited the narrowest and tallest-peaked distribution with the most pronounced leftward shift, achieving high-confidence predictions (entropy <0.2, probability <0.09 or >0.91) for ∼15.9% of compounds, very high confidence (entropy <0.3, probability <0.14 or >0.86) for ∼26.8%, and confident predictions (entropy <0.5, probability <0.21 or >0.79) for ∼45.1% (Figure S7 of the SI).^59^ Together, these results strongly affirmed the model’s robustness and its ability to provide consistent toxicity predictions across varied PM_2.5_ samples, highlighting its potential for broad application in profiling the toxic potential of organic compounds in diverse atmospheric environments.

In the context of analyzing a large number of PM_2.5_ samples and the diverse array of compounds that they contain, it is often a struggle to efficiently identify key toxic components using traditional methods. By integration of the predictive capabilities of ML models with specialized identification strategies targeting the aforementioned classes of compounds, screening efficiency is expected to improve significantly, thereby facilitating the rapid identification of key toxic components and further advancing related research.

### Environmental Implications

This study establishes an interpretable ML framework for predicting the toxicity of PM_2.5_ organic compounds by integrating curated A549 cytotoxicity data, customized MACCS fingerprints, and SHAP-based substructure analysis. By identifying aromatic rings and nitrogen-based functional groups (e.g., aromatic α,β-unsaturated ketones, aromatic amines, aromatic nitro compounds, and tertiary amines) as key toxicity-driving substructures—validated across 51 nontarget compounds spanning 13 chemical classes in Hong Kong PM_2.5_ samples—this framework provides important insights for identifying and controlling key PM_2.5_ toxicity components by significantly narrowing the search space from thousands of compounds to those containing critical toxicity-driving substructures. The robust performance on an independent data set of Nanjing PM_2.5_ samples confirms the model’s strong generalizability and demonstrates its broad applicability. This framework establishes a foundation for future advancements, such as the integration of regression-based models tailored to specific compound classes, including polycyclic aromatic compounds (PACs), to enhance the precision of toxicity predictions for aromatic rings and nitrogen-based functional groups.

Unlike previous studies that relied solely on computational predictions or labor-intensive assays, this ML framework balances model accuracy with real-world applicability by generating interpretable “structure–toxicity” rules. The integration of nontargeted chemical analysis and cytotoxicity experiments ensures that these predictions are grounded in empirical evidence, enhancing their credibility and practical utility. This approach overcomes major limitations of traditional PM_2.5_ toxicity assessments—such as time-consuming protocols, limited chemical coverage, and opaque decision-making—by enabling high-throughput and chemically explainable toxicity predictions. However, relying on A549 data may lead to some biological variability being overlooked. Furthermore, excluding inorganics (e.g., metals) and focusing on individual organic compounds rather than real-world complex mixtures could lead to underestimates of PM_2.5_ mixture toxicity. Incorporating diverse cell lines, *in vivo* models, and strategies to account for synergistic and antagonistic effects—such as mixture–toxicity modeling or multicompound inputs in ML frameworks—along with organic and inorganic concentrations, will help to refine the identification of key toxic components in ambient air PM_2.5_. Future research should also examine the long-term health effects of these components in diverse populations, clarifying the broader risks of PM_2.5_ exposure.

## Supplementary Material





## Notes

Data collection and filtering for a high-quality A549 cytotoxicity database (Text S1); quantitative uncertainty assessment (Text S2); SHAP approach (Text S3); PM_2.5_ sampling sites and methodology for Hong Kong and Nanjing, including sampling and pretreatment protocols (Text S4–S5); UHPLC–Orbitrap MS analysis (Text S6); nontargeted data processing workflow in Compound Discoverer (Text S7); auxiliary structural elucidation using MetFrag (Text S8); A549 cell toxicity assay protocol for IC_50_ determination (Text S9); A549 $\log_{10}\left(\frac{IC_{50}}{\mathrm{nM}}\right)$ distribution (Figure S1); distribution of Tanimoto similarity among 19,841 compounds in the A549 database (Figure S2); confusion matrix of the stacking ensemble model on the A549 cell toxicity training set (Figure S3) and distribution of high-toxicity (1) and low-toxicity (0) compounds predicted by the stacking ensemble model (Figure S4); prediction uncertainty for the Hong Kong (Figure S5) and Nanjing (Figure S7) PM_2.5_ data sets; predicted high-toxicity PM_2.5_ structures with fingerprint-bit annotations (Figure S6); cytotoxicity of confidence level 1 PM_2.5_ compounds (Table S8) (PDF)
Customized MACCS fingerprint definitions (Table S1), PM_2.5_ sample information (Table S2), tested-compound details (Table S3), model performance metrics across feature sets (Tables S4–S6), retention-time and characteristic-fragment comparison with standards (Table S7), and prediction results summary and validation against experimental values (Tables S9–S10) (XLSX)
