# Supplementary material for: Molecular Representation and Closed-Loop Validation for Toxicity Assessment of Organic Compounds in Ambient Air PM2.5
Source: Environ Sci Technol. 2026 Feb 25;60(13):10149–61. doi: 10.1021/acs.est.5c17667 (PMC13063816; doi:10.1021/acs.est.5c17667)
Supplement: Supplementary file 1 [file es5c17667_si_001.pdf]

Supporting Information

Molecular representation and closed-loop validation for toxicity assessment of organic compounds in ambient air $PM_{2.5}$

Yao Lu[a,b], Yangyang Wu[a,b], Xiangdong Li[a,b,*]

[a]Department of Civil and Environmental Engineering, The Hong Kong Polytechnic University, Hung Hom, Kowloon, Hong Kong

[b]The Hong Kong Polytechnic University Shenzhen Research Institute, Shenzhen, Guangdong 518057, China

*Corresponding author:

Xiangdong Li

Tel: (+852) 2766-6041

Fax: (+852) 2334-6389

Email: cexdli@polyu.edu.hk

Number of pages: 43

Number of Figures: 7

Number of Tables: 10 (9 of which are in the Excel file)

Table of Contents

Text S1. Data collection and filtering **S3-S4**

Text S2. Quantitative uncertainty assessment **S5-S6**

Text S3. SHapley Additive exPlanations (SHAP) approach **S7-S8**

Text S4. $PM_{2.5}$ sampling sites **S9-S10**

Text S5. $PM_{2.5}$ sampling methodology **S11**

Text S6. Sample pretreatment and UHPLC-Orbitrap MS analysis **S12-S13**

Text S7. Compound Discoverer analysis of nontargeted UHPLC-Orbitrap data **S14**

Text S8. *In silico* fragmentation tool MetFrag analysis **S15**

Text S9. A549 cell toxicity assay **S16**

Figure S1. Distribution of $\log_{10}$ (IC_{50} / nM) values for the A549 dataset **S17**

Figure S2. Distribution of Tanimoto similarity scores among 19,841 compounds in the A549 database **S18**

Figure S3. Confusion matrix of the Stacking Ensemble model on the A549 cell toxicity training set **S19**

Figure S4. Distribution of high toxicity and low toxicity compounds predicted by the Stacking Ensemble model **S20**

Figure S5. Distribution of prediction uncertainty across models on the independent Hong Kong $PM_{2.5}$ dataset (n = 387 compounds) **S21**

Figure S6. Chemical structures of $PM_{2.5}$ compounds predicted to have high toxicity, with fingerprint bits, by our Stacking Ensemble model **S22**

Figure S7. Distribution of prediction uncertainty across models on the independent Nanjing $PM_{2.5}$ dataset (n = 572 compounds) **S23**

Table S8. Cytotoxicity of Confidence Level 1 compounds in $PM_{2.5}$ **S24-S41**

References **S42-S43**

Text S1. Data collection and filtering

To build a database of cytotoxicity based on A549 cells, multiple publicly available databases were systematically examined. The primary databases assessed included the CompTox Chemicals Dashboard (https://comptox.epa.gov/dashboard), Tox21 (https://tox21.gov/tox21-library/), TOXRIC (https://toxric.bioinforai.tech/home), and PubChem (https://pubchem.ncbi.nlm.nih.gov/). However, searches in CompTox, Tox21, and TOXRIC yielded limited data on A549 cytotoxicity, with far fewer relevant entries compared to PubChem. Additionally, a literature search was conducted using Web of Science (https://www.webofscience.com) with the keywords "$PM_{2.5}$" and "A549," which yielded 504 relevant publications. The titles, authors, abstracts, and journal information of these articles were downloaded for further analysis. Utilizing VOS Viewer, a visualization tool, the co-occurrence of keywords was analyzed to identify prevalent research themes and hotspots within the "$PM_{2.5}$" and "A549" research areas. The resulting keyword co-occurrence map revealed a limited presence of terms related to specific chemical components, indicating a potential gap in the literature concerning detailed chemical composition analysis. Given that only 504 relevant publications were identified, the existing literature was deemed insufficient to establish a robust toxicity compound database solely through literature mining. Consequently, PubChem was selected as the primary data source due to its extensive and detailed repository of chemical and biological assay data. A keyword search for "A549" within PubChem yielded 16,887 entries, encompassing a total of 148,475 compounds associated with A549 cytotoxicity.

To ensure the quality and chemical plausibility of the dataset, a rigorous multi-step filtering process was implemented:

1) **IC_{50} Value Selection**: From the initial 148,475 compounds, an initial screening was performed based on the availability of half-maximal inhibitory concentration (IC_{50}) cytotoxicity data. This step retained only those compounds with reported IC_{50} values, reducing the dataset to 46,600 compounds. IC_{50} was selected as

the screening criterion due to its widespread use and reliability as a standard measure for assessing a compound's inhibitory effect on cell viability (Damiani et al., 2019).

2) **Molecular Characteristics Filtering**: Further refinement was conducted by retaining only compounds composed of carbon (C), hydrogen (H), nitrogen (N), oxygen (O), and sulfur (S) elements. This step excluded inorganic and metal-containing compounds, focusing the dataset on organic molecules relevant to $PM_{2.5}$ toxicity. Compounds were also filtered based on molecular weight, retaining those within the range of 50–900 Daltons (Da). This range corresponds to the molecular weights typically detected in organic compounds during nontargeted high-resolution mass spectrometry (HRMS) studies of $PM_{2.5}$ (Kourtchev et al., 2013). Additionally, the following structural constraints were enforced to ensure chemical feasibility: H/C ratio: 0.3–3.0, O/C ratio: 0.0–3.0, N/C ratio: 0.0–1.3, S/C ratio: 0.0–0.8, and DBE (double bond equivalent)/C ratio: 0.0–1.0 (Fuller et al., 2012; Wang et al., 2019). After this step, the dataset was further reduced to 25,077 compounds.

3) **Data Standardization**: To ensure consistency and comparability across the dataset, all IC_{50} values were standardized to nanomolar (nM) units and log10-transformed. For compounds with multiple IC_{50} measurements, the median value was selected as the representative value, and measurements with a standard deviation exceeding 1.5 log-mM were deemed unreliable and excluded (Peets et al., 2022).

Following these filtering and standardization steps, a final dataset comprising 19,841 high-quality compounds was obtained, establishing a robust foundation for subsequent machine learning model development.

Text S2. Quantitative uncertainty assessment

To rigorously evaluate the confidence of model predictions when applied to real-world ambient $PM_{2.5}$ samples, prediction uncertainty was quantified using the binary entropy of the predicted probability p (for belonging to the high-toxicity class, categorized as 1).

For a predicted probability $p \in [0, 1]$, the binary entropy H(p) is defined as

$$H(p) = -p \log_2(p) - (1-p) \log_2(1-p),$$

with edge cases handled by continuous extension ($\lim_{p \to 0} p \log_2 p = 0$). This metric ranges from 0 to 1 bit:

- H(p) = 0 indicates maximum certainty (p = 0 or p = 1),
- H(p) = 1 indicates maximum uncertainty (p = 0.5).

Due to the symmetry of the binary entropy function [H(p) = H(1−p)], this uncertainty measure is inherently class-agnostic and reflects overall prediction confidence regardless of the final assigned class.

Additionally, a practical confidence score was derived for each prediction as

$$\text{Confidence} = \max(p, 1-p).$$

This represents the probability assigned to the more likely class and also ranges from 0.5 (complete uncertainty) to 1.0 (complete certainty). The two metrics are directly related: low entropy corresponds to confidence close to 1.0, whereas entropy near 1 bit corresponds to confidence near 0.5.

For each detected compound in the Hong Kong $PM_{2.5}$ dataset (n = 387 compounds) and the independent Nanjing $PM_{2.5}$ dataset (n = 572 compounds), the predicted probability p of the high-toxicity class was obtained separately from the six base learners (Support Vector Machine, K-Nearest Neighbors, Decision Tree, Random Forest, XGBoost, and LightGBM) and from the final stacking ensemble model. Binary entropy was then computed individually for every compound–model pair.

The resulting uncertainty distributions were visualized using kernel density

estimation (KDE) with a Gaussian kernel. Bandwidth was automatically determined for each model using Scott's rule of thumb

$$h = 1.059 \times \hat{\sigma} \times n^{(-1/5)},$$

where $\hat{\sigma}$ is the standard deviation of the entropy values for that model and n is the number of compounds (387 or 572). This data-driven approach ensures optimal smoothness while preserving genuine features of each distribution (Figures S5 and S7).

Text S3. SHapley Additive exPlanations (SHAP) approach

In the global feature importance analysis, we calculated the average absolute SHAP values and the average algebraic SHAP values for each bit in the customized MACCS fingerprints. This allowed us to rank the importance of molecular substructures and identify features that significantly contribute to toxicity. Summary plots and bar charts can be used to effectively display the contribution levels of these features, with a focus on the top molecular substructures. By consulting the definitions of each bit in the customized MACCS fingerprints and utilizing the RDKit (https://www.rdkit.org/) tool, these high-SHAP-value features can be mapped back to specific molecular fragments, generating highlighted molecular structure diagrams that intuitively reveal the key chemical features and their influence mechanisms that the model focuses on during prediction.

The computations were performed on a system equipped with a 13th Gen Intel(R) Core(TM) i9-13900K CPU (3.00 GHz), 64.0 GB RAM, and an NVIDIA GeForce RTX 4070 GPU. To address the issue of high computational time (several hundred hours) required for calculating SHAP values when handling large datasets (training set containing 17,857 samples, 90% of the total) and complex models (such as SVM), we utilized clustering and stratified sampling techniques to improve efficiency. Specifically, we first partitioned the data into several clusters using MiniBatchKMeans(https://scikit-learn.org/stable/modules/generated/sklearn.cluster.MiniBatchKMeans.html). Within each cluster, a certain number of representative samples were selected using stratified sampling to ensure the representation of each category, along with a predefined number of background samples to enhance the accuracy and stability of SHAP explanations. Through these optimization techniques, the program efficiently computes SHAP values in large-scale and multi-algorithm environments. In this study, we selected a cluster number (k = 50), a sampling size of 468, and a background set size of 100. This workflow significantly improved computational efficiency by reducing the calculation time to 36 hours while maintaining the representativeness of the explanations. Ultimately, we obtained

comprehensive analysis results, including global feature importance rankings, SHAP values and molecular structure visualizations for representative samples, and statistics on high-frequency molecular substructures. These outcomes not only increased analysis efficiency by focusing on the most representative compounds but also facilitated a more intuitive understanding of the machine learning model's prediction rationale. Furthermore, this approach enabled a deeper exploration of the relationship between molecular features and toxicity prediction, providing an efficient and scientifically meaningful framework for model interpretation.

Text S4. $PM_{2.5}$ sampling sites

Hong Kong $PM_{2.5}$ samples were collected from five sites. Two of these sites were located at The Hong Kong Polytechnic University (PolyU), while the remaining three were managed by the Hong Kong Environmental Protection Department (HKEPD).

1) PolyU-Ambient (22.31°N, 114.18°E)

The PolyU-ambient site is situated on the sixth-floor roof garden of Block Z at PolyU. This location features limited vegetation and serves as a representative general environmental setting with minimal direct influence from nearby traffic sources. The rooftop garden provides a typical urban environment, allowing for the assessment of ambient air quality under relatively stable conditions.

2) PolyU-Roadside (22.31°N, 114.18°E)

The PolyU-roadside site is located adjacent to the heavily trafficked Cross-Harbour Tunnel, a major transportation hub connecting Hong Kong Island and Kowloon. This area is characterized by high vehicular emissions due to significant daily traffic volumes, especially during peak hours. The surrounding infrastructure includes narrow roads and dense buildings, creating an urban canyon effect that exacerbates the accumulation of air pollutants such as $PM_{2.5}$. This site effectively captures the impact of traffic-intensive environments on local air quality.

3) Kwai Chung (22.37°N, 114.11°E)

The Kwai Chung site is located in a mixed industrial-residential area along Kwai Chung Road. This environment is influenced by nearby industrial facilities, major road networks, and residential buildings, resulting in elevated levels of $PM_{2.5}$ and other pollutants from both industrial emissions and vehicular traffic. The site provides valuable data on the combined effects of industrial activities and dense urban infrastructure on air quality.

4) Tung Chung (TUC, 22.28°N, 113.94°E)

Situated at Fu Tung Street in Tung Chung, this site represents a coastal suburban environment on Lantau Island. The area is predominantly residential with adjacent commercial zones and is significantly influenced by prevailing sea breezes, which can disperse and dilute airborne pollutants. The coastal location allows for the assessment

of background air quality conditions and the impact of marine influences on $PM_{2.5}$ concentrations.

5) Southern District (SOU, 22.23°N, 114.15°E)

The Southern District site is located in Aberdeen, characterized by a coastal residential environment with low-density housing and proximity to the coastline. Serving as a background monitoring station, this site experiences minimal direct emissions from industrial or high-traffic sources. The coastal setting provides a baseline for ambient air quality, allowing for comparisons with more polluted urban and industrial sites.

These five sampling locations collectively encompass a diverse range of environmental settings, including general ambient conditions, traffic-intensive areas, mixed industrial-residential zones, coastal suburban regions, and coastal background sites.

Nanjing $PM_{2.5}$ samples were collected from two key sites.

1) Xuanwu urban site (32.07° N, 118.79° E)

Located on a rooftop in central Nanjing's Xuanwu District, this site represents a dense urban environment with significant influence from traffic, industrial activities, and commercial operations.

2) Lishui rural site (31.55° N, 119.07° E)

Situated in an agricultural area in Lishui District, this site characterizes a rural background setting with minimal local pollution sources. It is primarily influenced by regional transport and agricultural activities, resulting in lower $PM_{2.5}$ levels and a chemical profile reflective of regional conditions. This site provides baseline data for evaluating the incremental pollution from urbanization.

These sites collectively offer a clear urban-rural gradient, facilitating investigations into spatial disparities in $PM_{2.5}$ composition and toxicity within the Nanjing region.

Text S5. $PM_{2.5}$ sampling methodology

Two types of high-volume air samplers were utilized, with the TISCH TE-6070 High-Volume Air Sampler (TISCH Environmental Inc.) deployed at Tung Chung and Kwai Chung, and the Mingya High-Volume Sampler (Guangzhou Mingya Environmental Technology Co., Ltd.) used at the remaining locations. The sampling flow rate was maintained at 40–43 CFM (approximately 1 m^3/min), with site-specific flow rates measured as 1.134 m^3/min at Tung Chung, 1.126 m^3/min at Kwai Chung, and 0.999 m^3/min at the remaining sites. Each sampling session lasted for 24 hours. Pallflex® Tissuquartz™ quartz filters (8 × 10 inches, PALL) were used for particle collection. Prior to sampling, the filters were baked at 550°C for 4 hours to remove organic contaminants and equilibrated under controlled conditions (15–25°C and 50±5% relative humidity) for 24 hours. The filters' initial weights were recorded with a precision of 0.1 mg, and the coarse side of each filter was placed facing upward to ensure uniform particle collection.

On-site sampling was conducted in strict accordance with standard operating procedures. After each sampling session, filters were carefully removed, folded with the sample side inward, wrapped in aluminum foil, and labeled with comprehensive details, such as the sampling date, flow rate, duration, and operator information. Tools, including tweezers and scissors, were thoroughly cleaned with 70–80% ethanol and allowed to air dry before reuse. Filter holders were cleaned after every session to minimize the risk of contamination. To ensure data quality, multiple quality control measures were implemented. These included batch blank filters, seasonal field blanks for background correction, and regular maintenance of the samplers. The inlets and impactors were cleaned periodically, and flow meters were calibrated to ensure accuracy and consistency.

Post-sampling, filters were equilibrated again under controlled conditions for 24 hours before weighing. If the weight variation was within 1 mg, the average value was used; otherwise, additional equilibration and re-weighing were performed. Filters were sealed in plastic bags and stored at -20°C to prevent degradation.

Text S6. Sample pretreatment and UHPLC-Orbitrap MS analysis

$PM_{2.5}$ quartz filters were placed into pre-cleaned glass extraction containers, and an appropriate amount of methanol (MeOH, chromatography grade, Honeywell, Charlotte, NC) was added for organic solvent extraction. The extraction process involved ultrasonic bath treatment at room temperature for 30 minutes, repeated three times to ensure thorough extraction of organic compounds. After extraction, the samples were evaporated under a gentle nitrogen stream. The dried extracts were then reconstituted in a 1:1 (v/v) mixture of methanol and ultrapure water, filtered through a 0.22 μm PTFE membrane, and transferred to glass vials prepared for UHPLC-Orbitrap MS analysis. Throughout the extraction process, blank samples and replicates were included to ensure contamination control and evaluate the reproducibility of the procedure.

For Hong Kong $PM_{2.5}$ samples, UHPLC separation was conducted on an ACQUITY UPLC BEH C_{18} column (2.1 × 100 mm, 1.7 μm, Waters Corporation) at a flow rate of 0.2 mL/min. The mobile phases consisted of 0.1% formic acid in ultrapure water (phase A) and 0.1% formic acid in methanol (phase B). The gradient elution program was as follows: 0–3 minutes, 0%–3% B; 3–25 minutes, 3%–50% B; 25–43 minutes, 50%–90% B; 43–48 minutes, 90%–3% B; and held at 3% B for 6 minutes. Orbitrap mass spectrometry was performed in both positive (ESI^+) and negative (ESI^-) electrospray ionization modes, scanning a mass-to-charge ratio (*m/z*) range of 50–900. Data acquisition utilized data-dependent collision-induced dissociation multistage mass spectrometry (MS^n, where n = 1 to 4). MS parameters included a static spray voltage, sheath gas flow rate of 30 arbitrary units, auxiliary gas flow rate of 20 arbitrary units, sweep gas flow rate of 0, and both ion transfer tube and vaporizer temperatures set to 300°C. The Orbitrap resolution was set to 120,000 for MS^1 scans and 30,000 for MS/MS acquisition.

For Nanjing $PM_{2.5}$ samples, the mobile phases consisted of 0.1% formic acid in ultrapure water (Phase A) and 0.1% formic acid in acetonitrile (Phase B). A linear gradient elution was applied as follows: 5% Phase B held for the first minute,

increased to 70% Phase B at 8 minutes, further increased to 99% Phase B at 12 minutes, maintained for 2 minutes, and then rapidly returned to 5% Phase B at 14.1 minutes, where it was held for an additional 2.9 minutes to ensure column re-equilibration. The flow rate was maintained at 0.3 mL/min throughout the analysis. This gradient program enabled effective separation of analytes with varying polarities while maintaining good peak shape and resolution. Mass spectrometric detection was performed in both positive and negative ion modes using a heated electrospray ionization (H-ESI) source. The spray voltage was set to 3500 V in positive mode and -3500 V in negative mode. Other shared parameters included sheath gas flow rate (45 Arb), auxiliary gas flow rate (10 Arb), sweep gas flow rate (2 Arb), vaporizer temperature (320 °C), and ion transfer tube temperature (300 °C). Full-scan MS data were acquired in the Orbitrap analyzer at a resolution of 120,000 (at *m/z* 200), with a scan range of *m/z* 70–1000. Data-dependent acquisition (DDA) was enabled with dynamic exclusion (4 s), isotope exclusion on, and stepped collision energy (20%, 30%, 50%) for HCD fragmentation. The Automatic Gain Control (AGC) target was set to standard with a maximum injection time of 54 ms. This analytical configuration facilitated the in-depth profiling and structural elucidation of a diverse range of organic compounds present in $PM_{2.5}$.

Text S7. Compound Discoverer analysis of nontargeted UHPLC-Orbitrap data

The obtained UHPLC-Orbitrap MS data were processed using the Compound Discoverer software (Thermo Fisher Scientific, Waltham, MA, USA), which integrated built-in databases such as ChemSpider, MzCloud, mzVault, Mass Lists, and BioCyc for molecular structure and fragmentation pattern matching, thereby improving the precision of compound identification.

During the nontargeted peak detection and compound identification process, peaks with a signal-to-noise ratio (S/N) of ≥3 were initially selected, restricting their molecular formulas to the range $C_{1–39}H_{1–72}O_{0–20}N_{0–7}S_{0–4}$. For peaks detected in procedural blank samples (baked blank filters), only those exceeding the procedural blank by at least tenfold were retained. Using the "Group Compounds" module, the detected peaks were grouped, combining signals attributed to the same compound. The "Mark Background Compounds" module was then employed to label background compounds, enabling the elimination of interfering peaks in subsequent analyses. Next, the peaks were matched against multiple databases, including ChemSpider, MzCloud, mzVault, Mass Lists, and BioCyc, to compare molecular structures and fragmentation patterns, thereby enhancing the accuracy and reliability of compound identification. During the screening process, strict criteria were applied to ensure high-quality identification results. These criteria included non-empty compound names, mass deviations within the range of -5 ppm to +5 ppm, complete matching statuses from multiple databases, the presence of MS^2 data, MzCloud and mzVault best match scores of ≥ 80.00, and peak ratings of ≥ 3.00.

Finally, Compound Discoverer presented the compound identification results in a tabular format. These results included each compound's name, molecular formula, mass deviation, fragmentation match scores, peak abundance, and source database information.

Text S8. *In silico* fragmentation tool MetFrag analysis

During the chemical structure elucidation process, the *in silico* fragmentation tool MetFrag was employed as an auxiliary tool to identify nontarget compounds (Ruttkies et al., 2016). By predicting the fragmentation patterns of compounds and comparing these predictions with experimentally obtained MS^2 spectra, the alignment between observed and theoretical fragments was evaluated. High matching scores and a sufficient number of characteristic fragments significantly improved the robustness of structural elucidation. To optimize the fragment generation process, varying levels of higher-energy collisional dissociation (HCD) were utilized to acquire strong parent ion signals and generate detailed fragmentation spectra. Additionally, isotopic pattern recognition was employed to confirm the presence of key elements within the molecular structure, further validating structural characteristics. Retention time prediction models were integrated into the elucidation workflow to ensure agreement between experimental and theoretical retention times. Comparison with reference standards was also conducted to confirm compound identities conclusively.

By comprehensively applying fragmentation matching, isotopic pattern analysis, retention time calibration, and standard comparisons, highly accurate and reproducible preliminary identification of compounds at Confidence Level 3 (CL 3) was achieved (Schymanski et al., 2014).

Text S9. A549 cell toxicity assay

A549 cells were cultured in F-12K medium supplemented with 10% fetal bovine serum (FBS) and 1% penicillin-streptomycin. Cultures were maintained at 37°C in a humidified incubator with 5% carbon dioxide (CO_2) (Zou et al., 2016).

For toxicity assessment, cell viability was evaluated using the MTS assay, which quantified the IC_{50} of the tested compounds. Log-phase A549 cells were diluted to approximately 10,000 cells per well and seeded into 96-well plates. Each experimental condition was performed in triplicate. Additionally, blank wells containing only culture medium and negative control wells were included to ensure the validity and reproducibility of the assay.

Prior to compound exposure, seeded cells were incubated for 24 hours at 37°C with 5% CO_2 to allow for adhesion and stabilization. Compound solutions at varying concentrations were then prepared, and each well received 100 μL of the prepared compound solution, followed by a 48-hour incubation under the same conditions.

After 48 hours of compound exposure, 20 μL of the MTS working solution, prepared according to the manufacturer's protocol, was added to each well. Plates were incubated for an additional 2 to 4 hours to allow viable cells to metabolize the MTS reagent, which produced a measurable colorimetric response. Optical density (OD) at 490 nm was measured using a microplate reader to assess cell viability.

IC_{50} values were calculated by plotting the percentage inhibition of cell viability against the logarithm of compound concentrations, and generating concentration-response curves. Nonlinear regression analysis was performed to determine the concentration of each compound that resulted in a 50% reduction in cell viability.

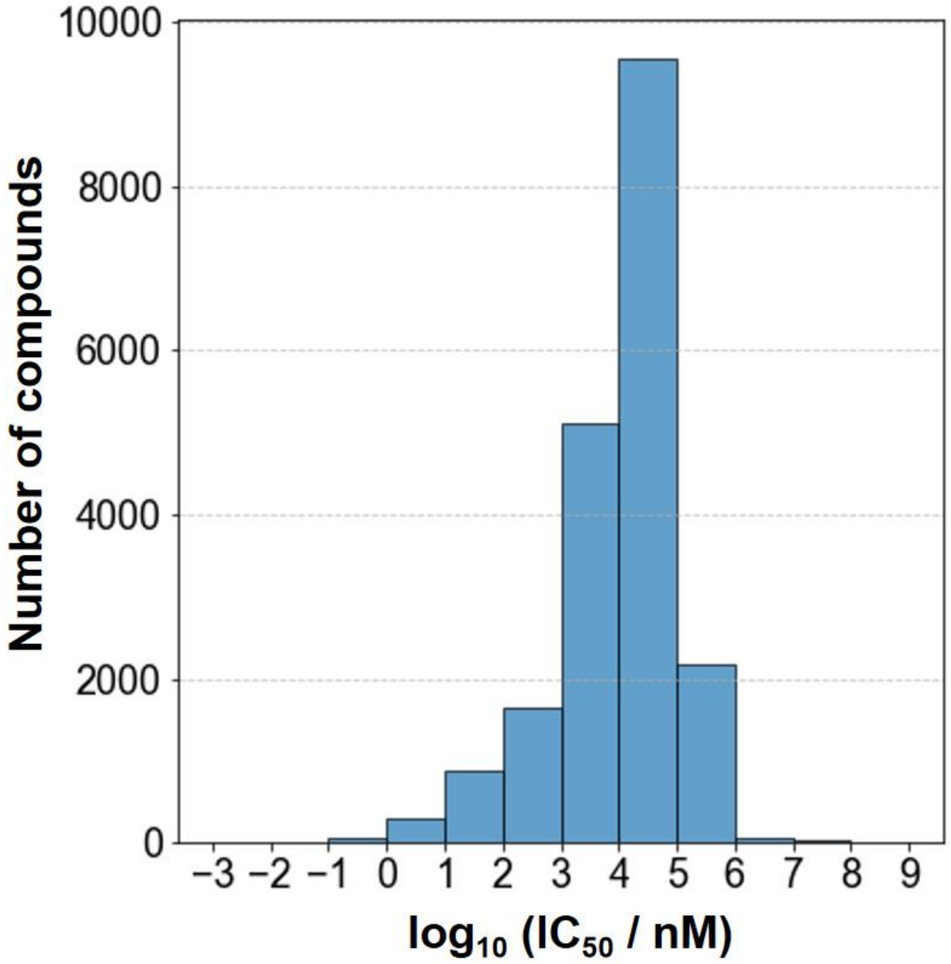

Figure S1. Distribution of $\log_{10}$ (IC_{50} / nM) values for the A549 dataset, which includes 19,841 compounds. Compounds are classified based on $\log_{10}$ (IC_{50} (nM)), with $\log_{10}$ (IC_{50} / nM) ≥ 4 classified as low toxicity (0) (11,781 compounds) and $\log_{10}$ (IC_{50} / nM) < 4 classified as high toxicity (1) (8,060 compounds).

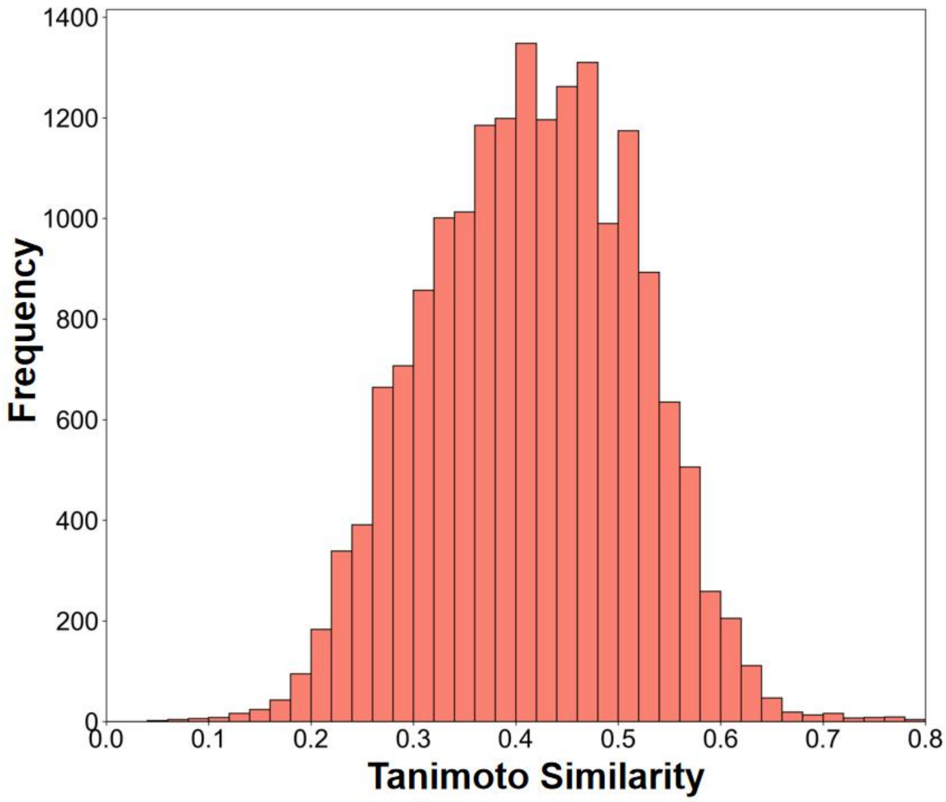

Figure S2. Distribution of Tanimoto similarity scores among 19,841 compounds in the A549 database.

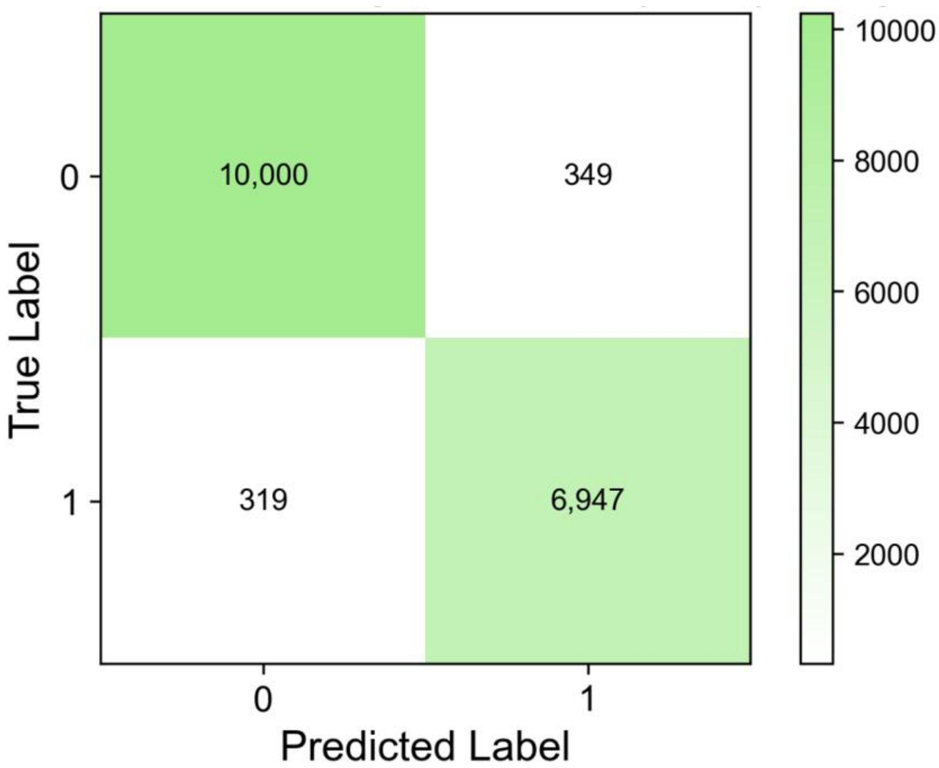

Figure S3. Confusion matrix of the Stacking Ensemble model on the A549 cell toxicity training set.

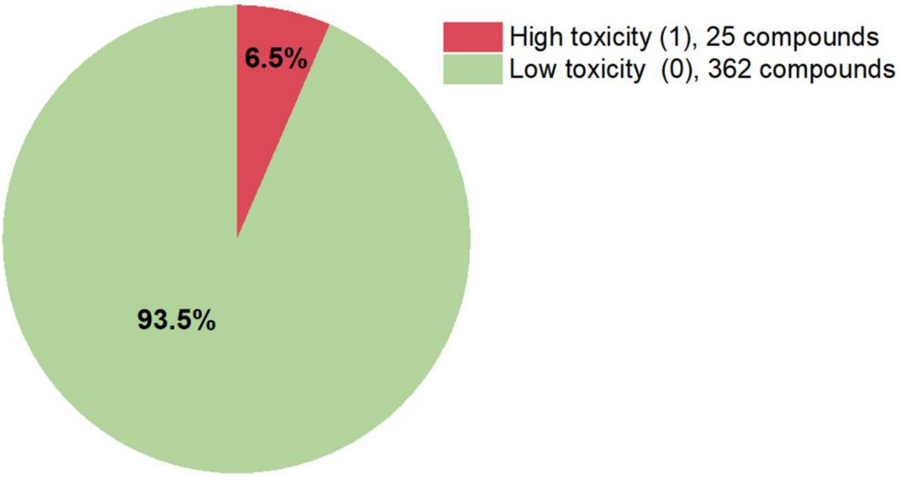

Figure S4. Distribution of high toxicity (1) and low toxicity (0) compounds predicted by the Stacking Ensemble model.

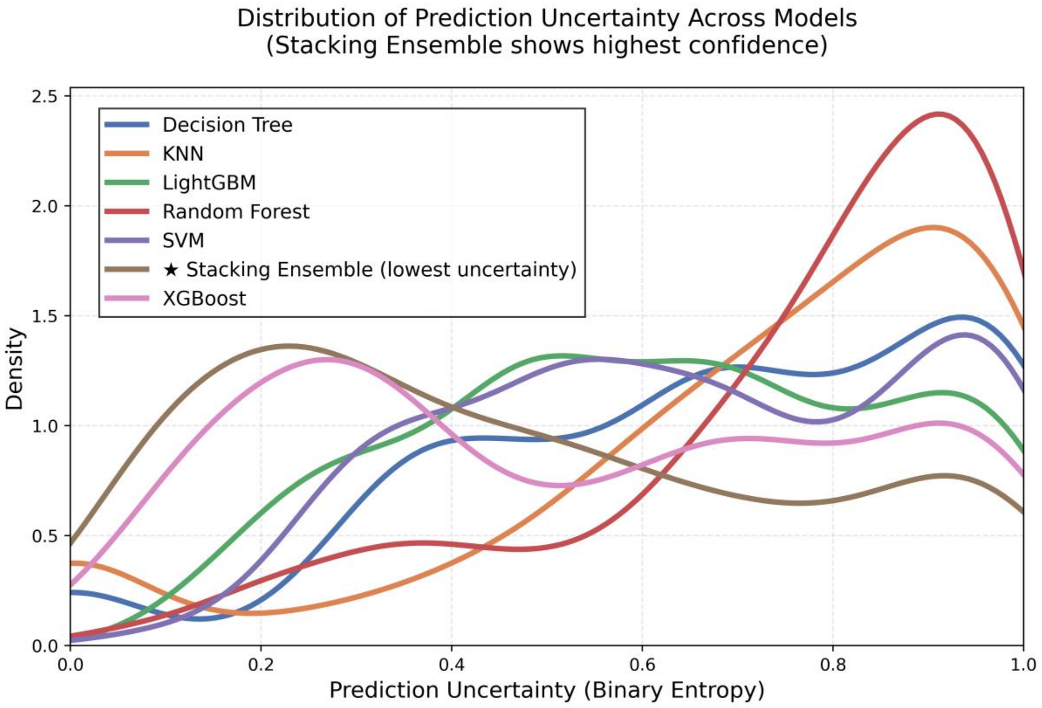

Figure S5. Distribution of prediction uncertainty across models on the independent Hong Kong $PM_{2.5}$ dataset (n = 387 compounds).

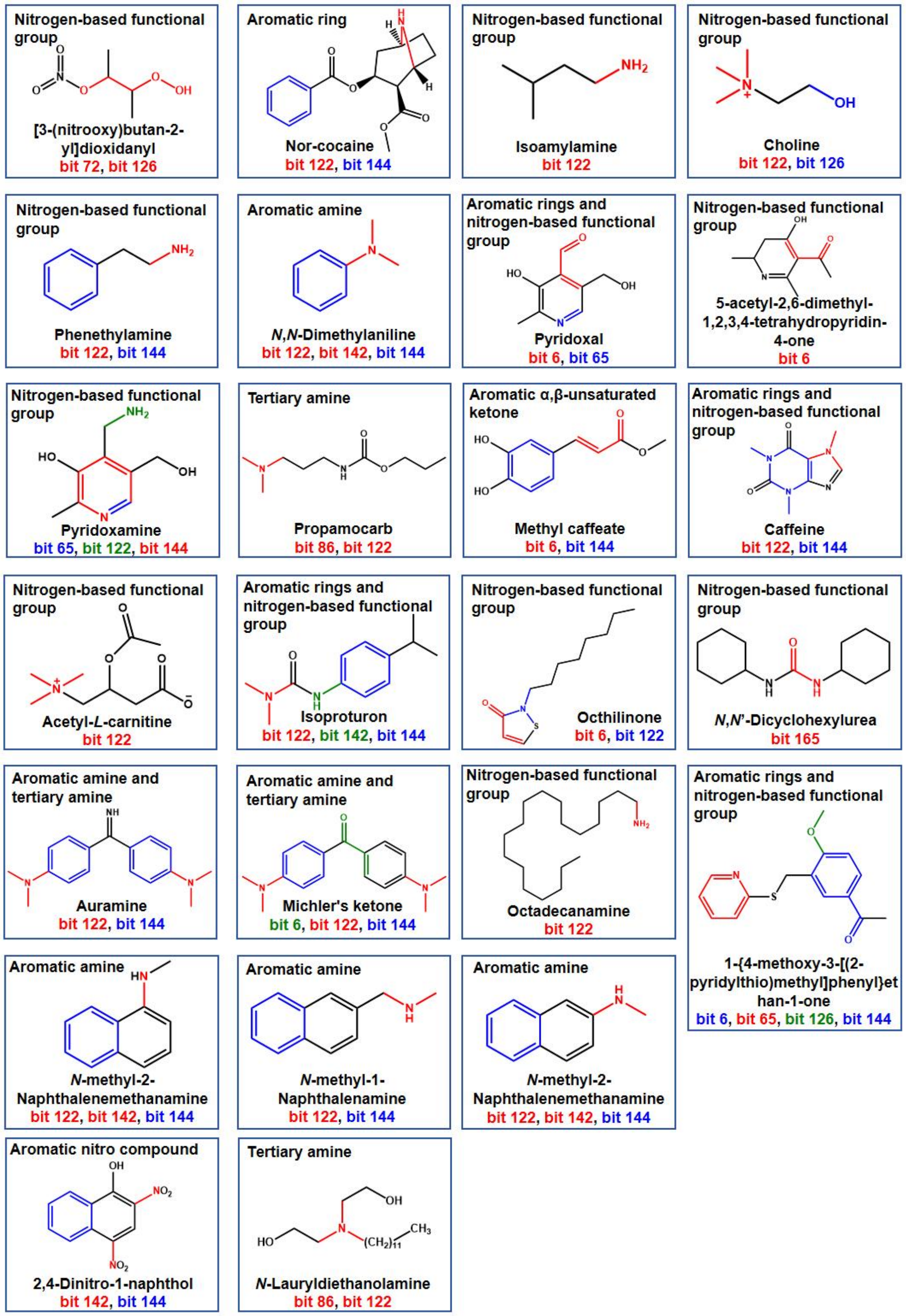

Figure S6. Chemical structures of compounds in $PM_{2.5}$ predicted to have high toxicity (1) by our Stacking Ensemble model, with fingerprint bits corresponding to the top 20 representative features identified based on SHAP values.

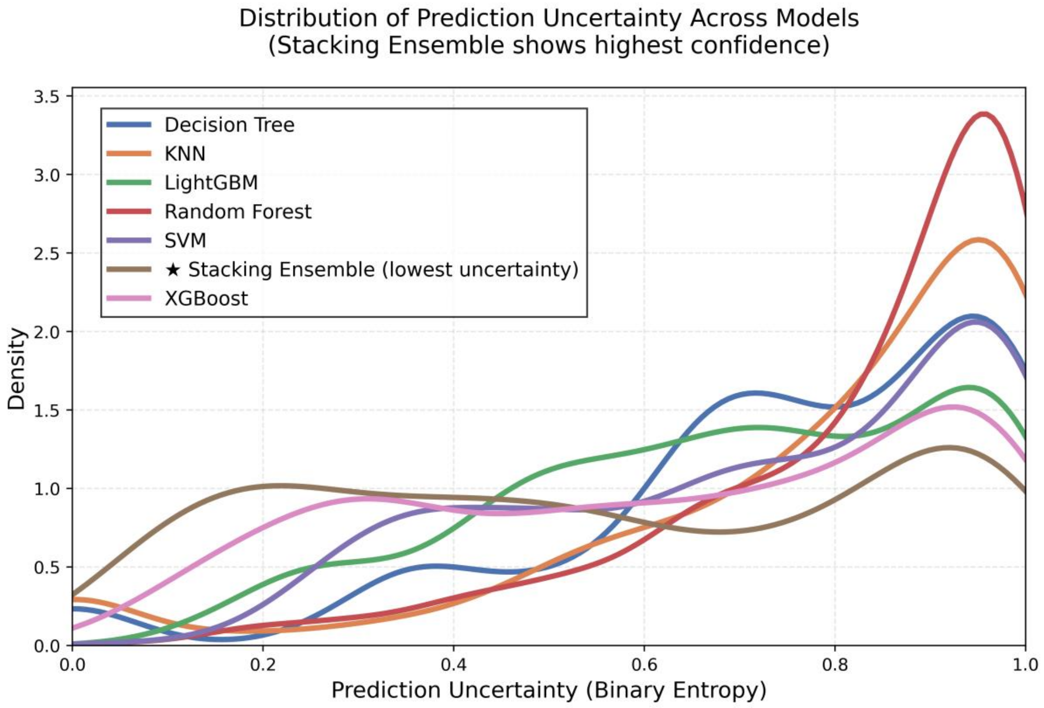

Figure S7. Distribution of prediction uncertainty across models on the independent Nanjing $PM_{2.5}$ dataset (n = 572 compounds).

Table S8. Cytotoxicity of Confidence Level 1 compounds in $PM_{2.5}$ (48-hour exposure)

$PM_{2.5}$-associated compounds	Concentration-effect curve	Cytotoxicity (IC_{50}, M)	Toxicity Level
**Alcohols and ethers (n = 4)**			
Diethylene glycol monobutyl ether	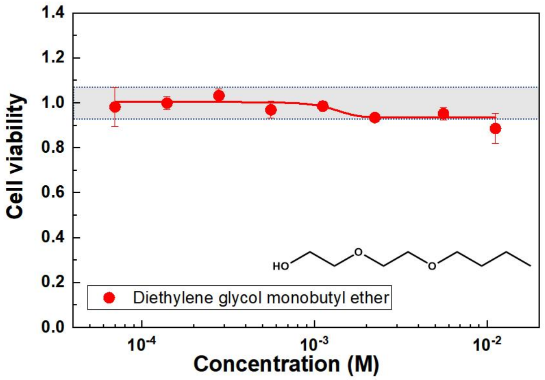	ND [a]	0
Levoglucosan	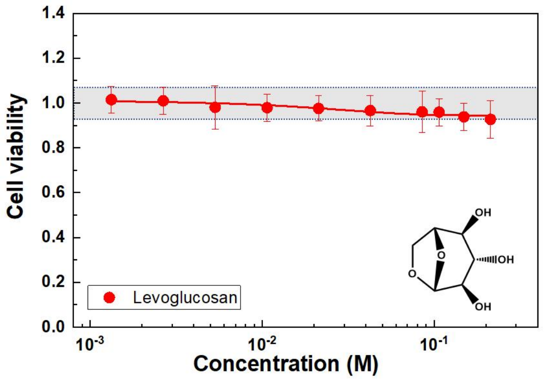	ND	0

meso-Erythritol	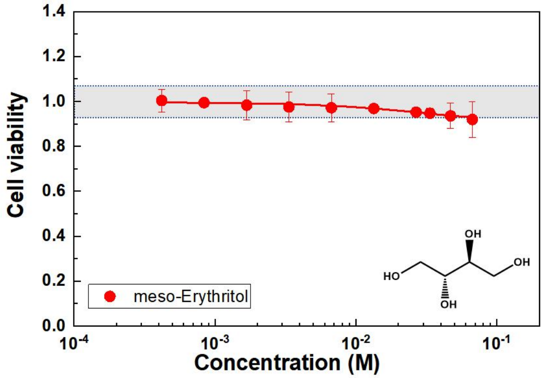	ND	0
Triethylene glycol	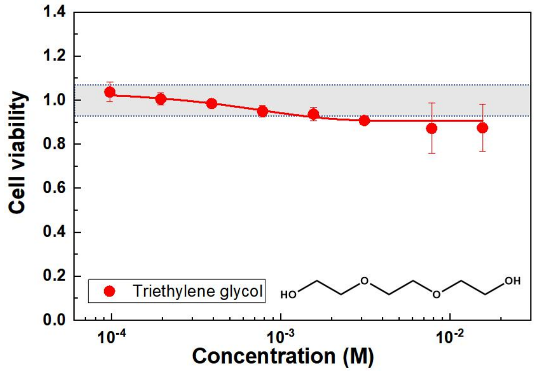	ND	0
**Aldehydes and ketones (n = 5)**			
Benzophenone	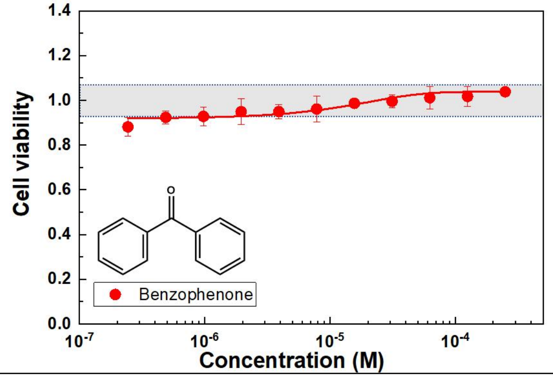	ND	0

Isophorone	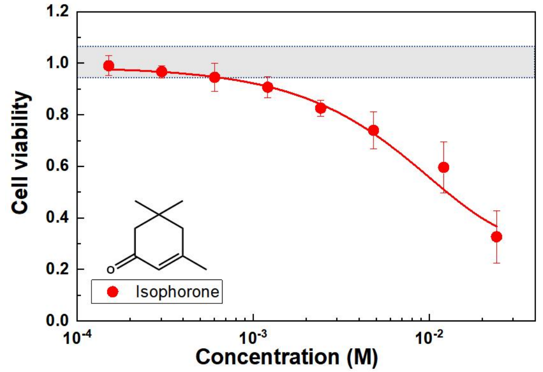	$(1.26 \pm 0.07) \times 10^{-2}$	0
3-Methoxybenzaldehyde	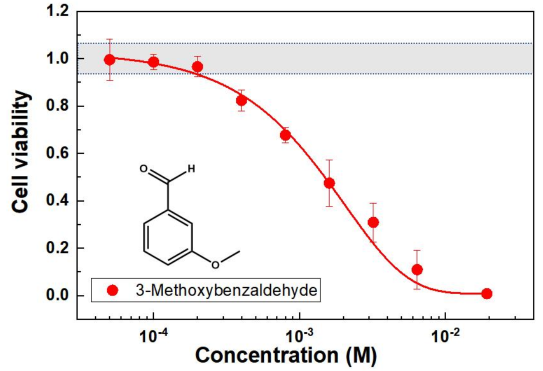	$(1.50 \pm 0.08) \times 10^{-3}$	0
Syringaldehyde	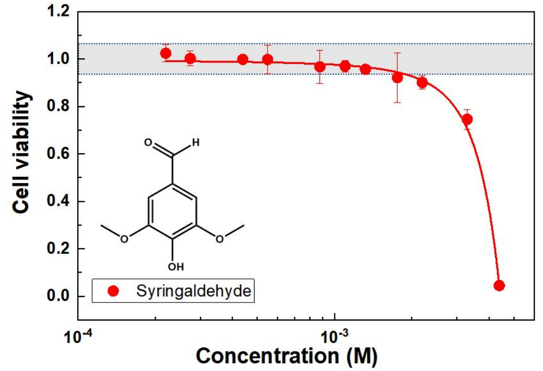	$(3.78 \pm 0.07) \times 10^{-3}$	0

Vanillin	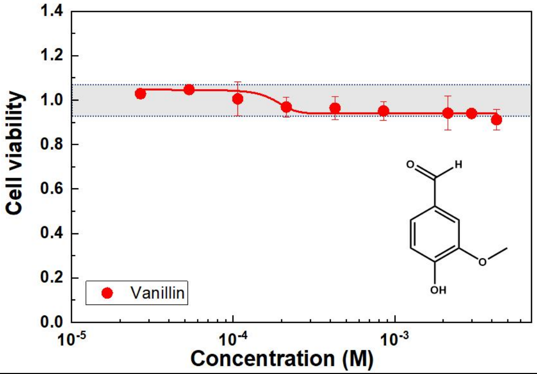	ND	0
**Amides (n = 4)**			
N,*N*-dibutylformamide	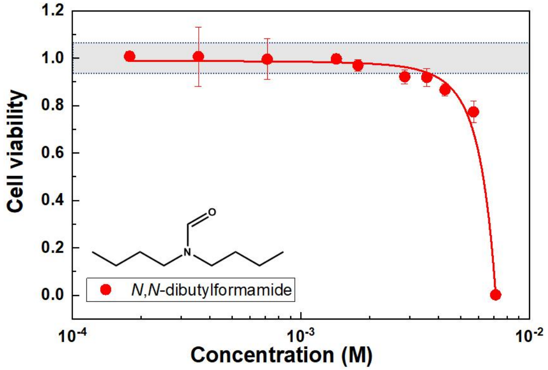	$(6.27 \pm 0.26) \times 10^{-3}$	0
N-(4-Ethoxyphenyl)acetamide	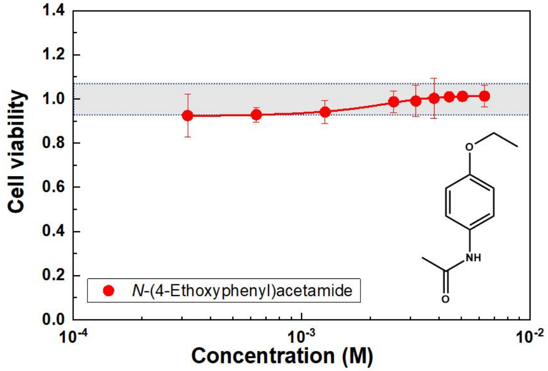	ND	0

N-(4-Hydroxyphenyl)acetamide	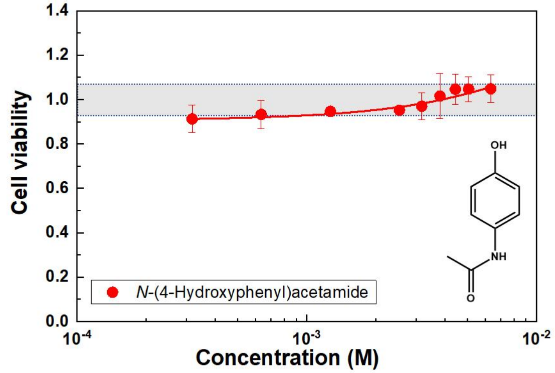	ND	0
2-Pyrrolidinone	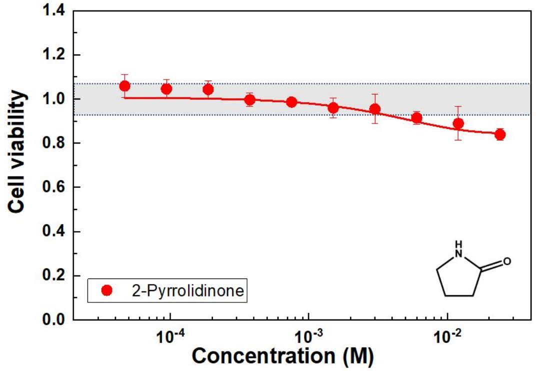	ND	0
**Amines (n = 10)**			
3-Aminofluoranthene	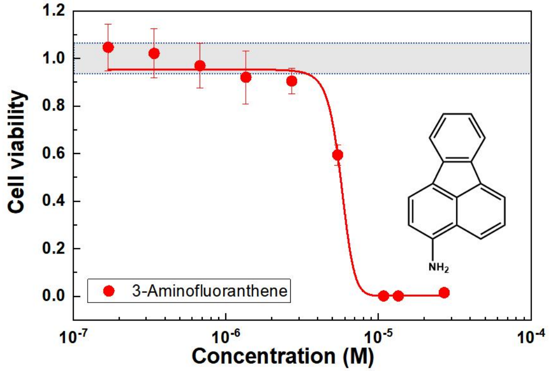	$(5.62 \pm 0.32) \times 10^{-6}$	1

Cyclohexylamine	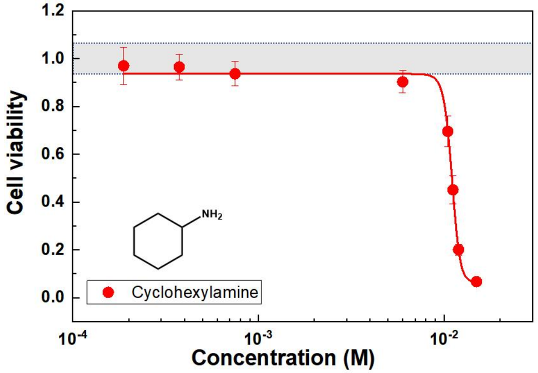	(1.11 ± 0.05) × 10^{-2}	0
Dicyclohexylamine	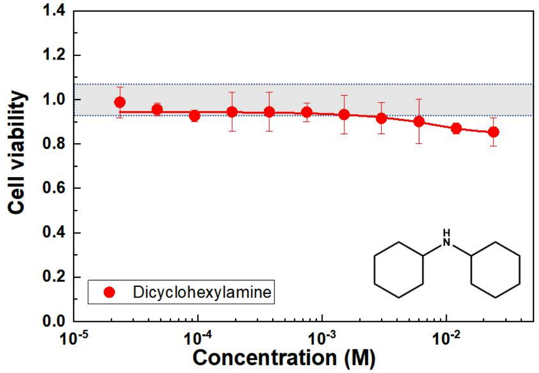	ND	0
4,4’-Diaminodiphenylmethane	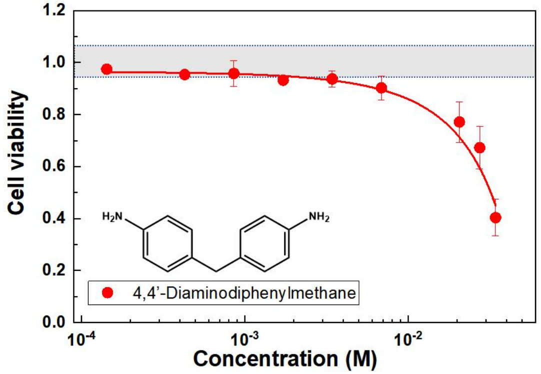	(3.19 ± 0.13) × 10^{-2}	0

1-Naphthalenamine	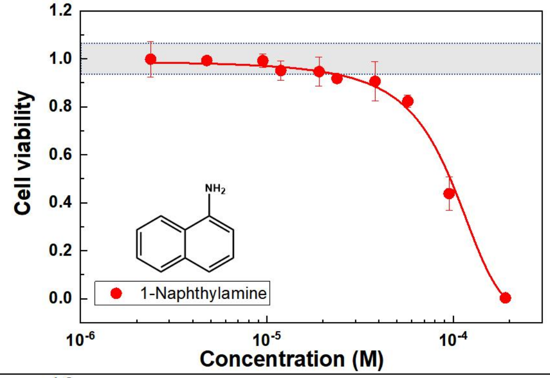	$(9.63 \pm 0.49) \times 10^{-5}$	0
2-Naphthalenamine	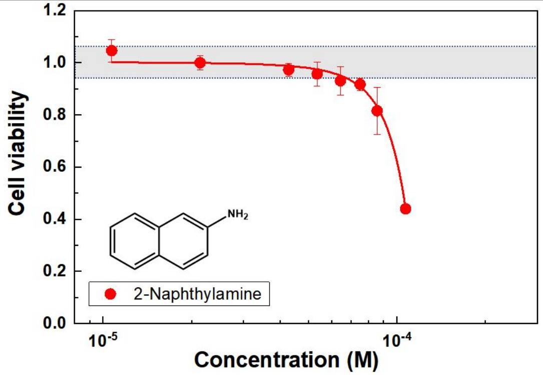	$(1.05 \pm 0.04) \times 10^{-4}$	0
N-Methyl-1-naphthalenamine	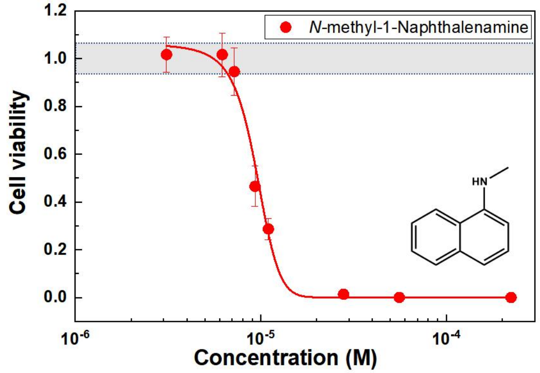	$(9.62 \pm 0.41) \times 10^{-6}$	1

N-Methyl-2-naphthalenamine	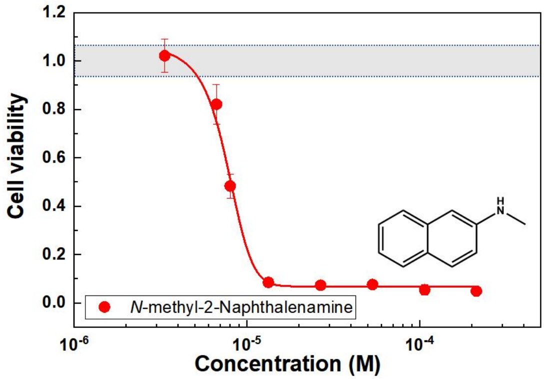	$(8.07 \pm 0.44) \times 10^{-6}$	1
N-Methyl-2-naphthalenemethanamine	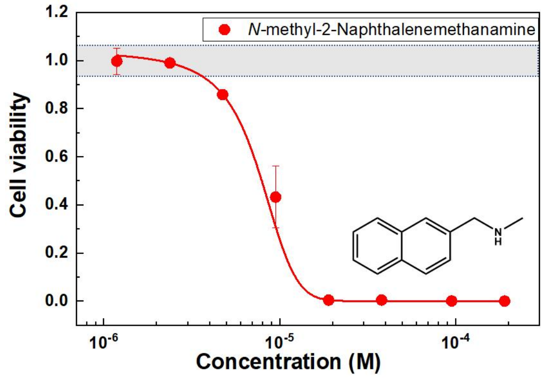	$(7.90 \pm 0.19) \times 10^{-6}$	1
N-Lauryldiethanolamine	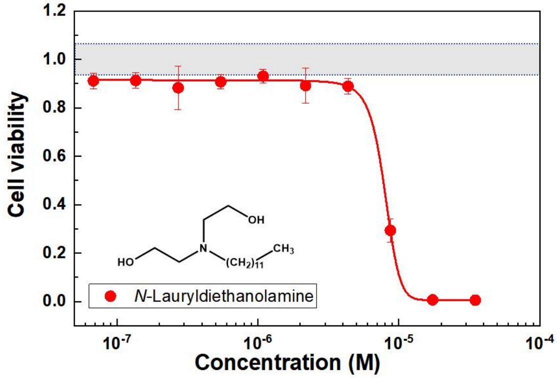	$(7.75 \pm 0.13) \times 10^{-6}$	1

**Anhydride (n = 2)**			
Phthalic anhydride	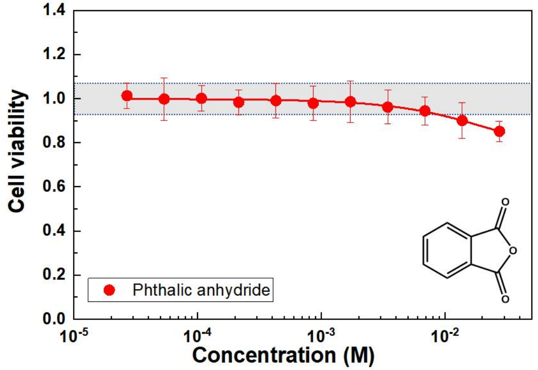	ND	0
1,8-Naphthalic anhydride	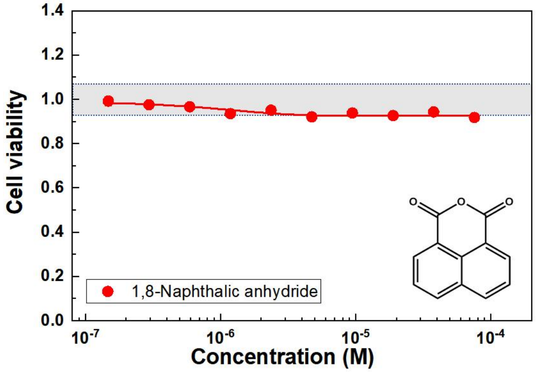	ND	0
**Carboxylic acids (n = 4) and amino acids (n = 1)**			
Azelaic acid	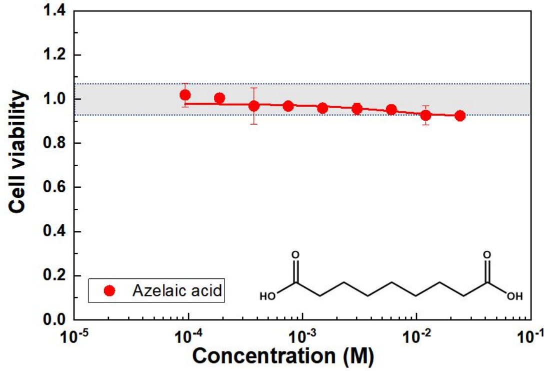	ND	0

Fumaric acid	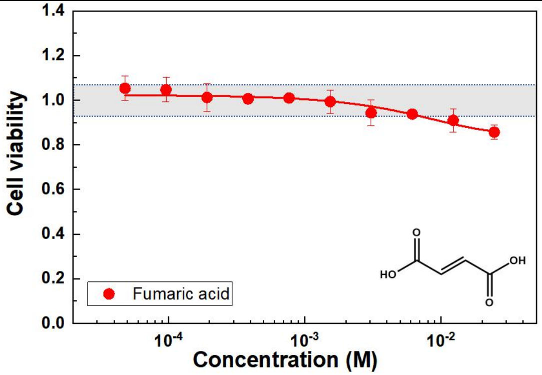	ND	0
Phthalic acid	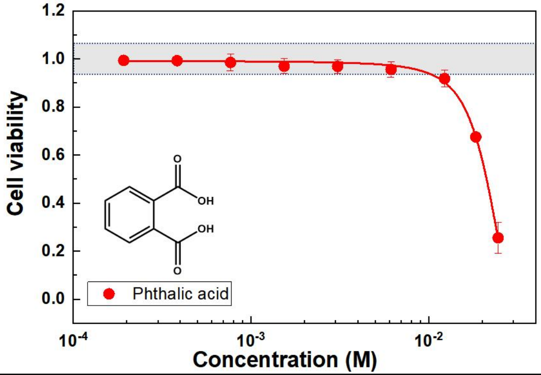	(2.12 ± 0.06) × 10^{-2}	0
Succinic acid	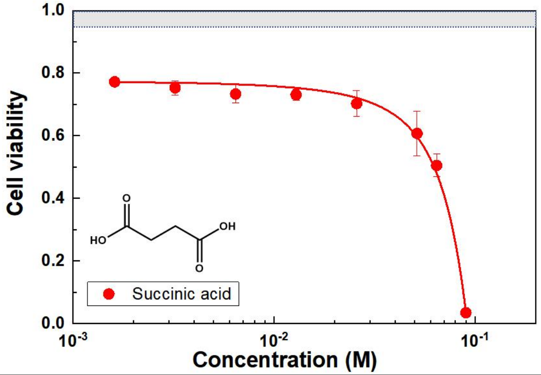	(6.26 ± 0.13) × 10^{-2}	0

L-phenylalanine	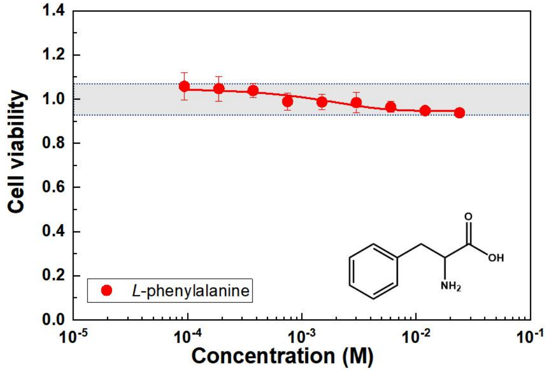	ND	0
**Carboxylic esters (n = 2) and phthalates (n = 1)**			
Dimethyl glutarate	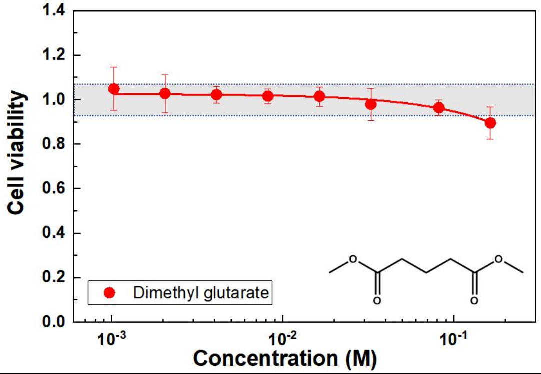	ND	0
Methylparaben	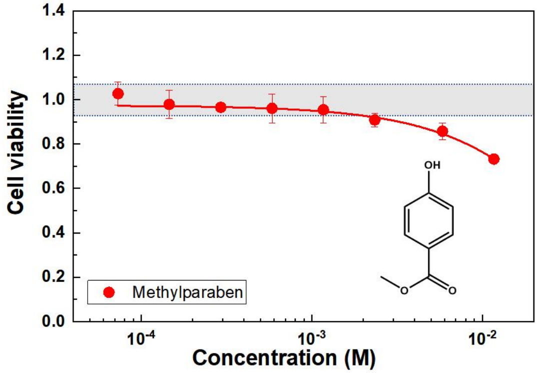	ND	0

Dibutyl phthalate	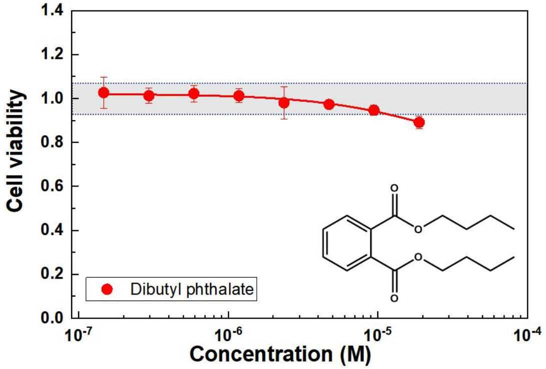	ND	0
**Nitrogen-heterocyclic compounds (n = 7)**			
4,4’-Bipyridine	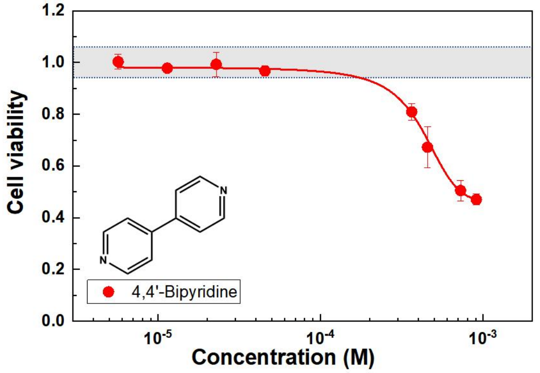	$(7.31 \pm 0.26) \times 10^{-4}$	0
2,6-Dimethylpyrazine	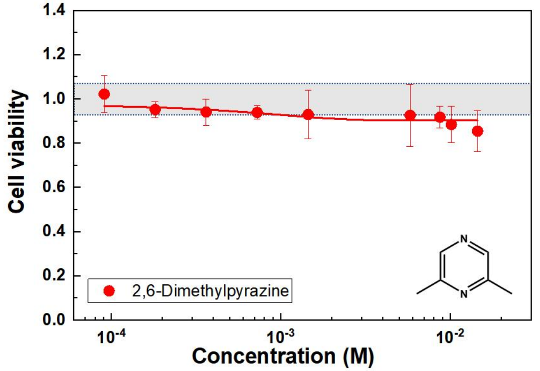	ND	0

2-Hydroxynicotinic acid	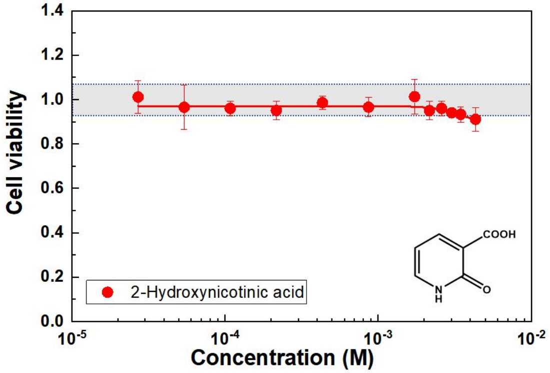	ND	0
2-Methylbenzimidazole	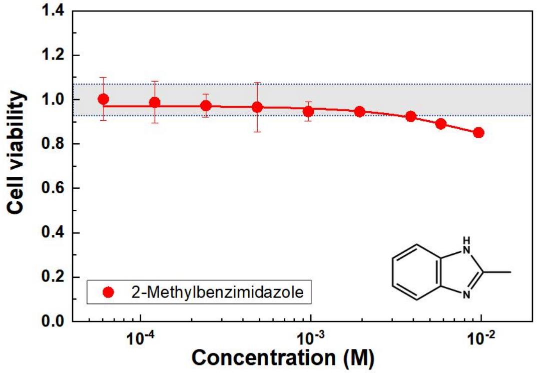	ND	0
7-Nitroindole	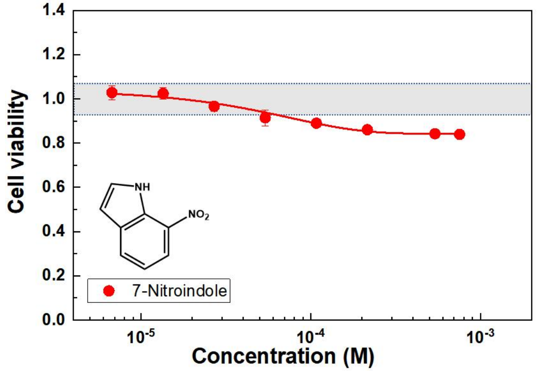	ND	0

Picolinic acid	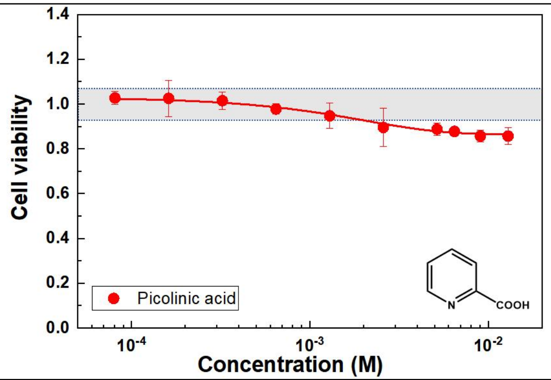	ND	0
Quinoline	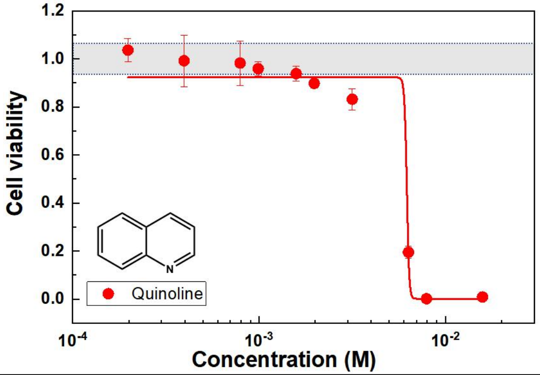	$(6.20 \pm 0.25) \times 10^{-3}$	0
**Nitro-compounds (n = 6)**			
2,4-Dinitro-1-naphthol	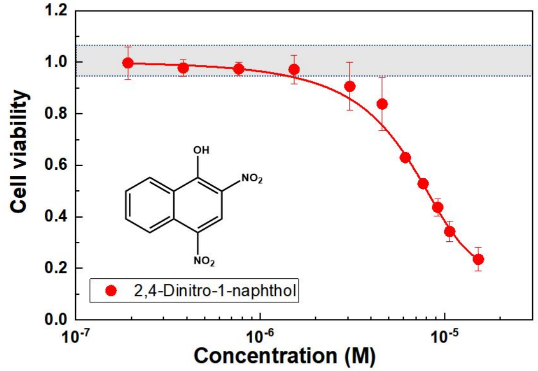	$(8.03 \pm 0.38) \times 10^{-6}$	1

3-Methyl-4-nitro-phenol	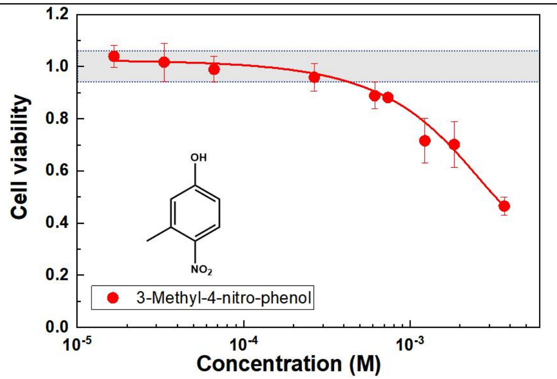	$(3.21 \pm 0.17) \times 10^{-3}$	0
4-Nitro-1-naphthol	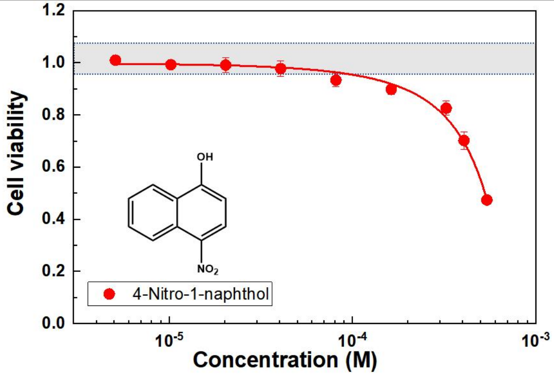	$(5.27 \pm 0.11) \times 10^{-4}$	0
4-Nitrophenol	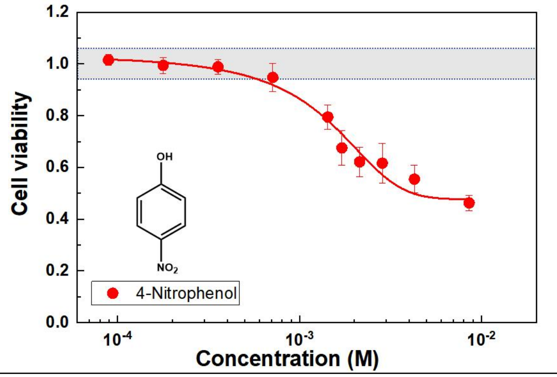	$(4.19 \pm 0.02) \times 10^{-3}$	0

5-Nitrosalicylic acid	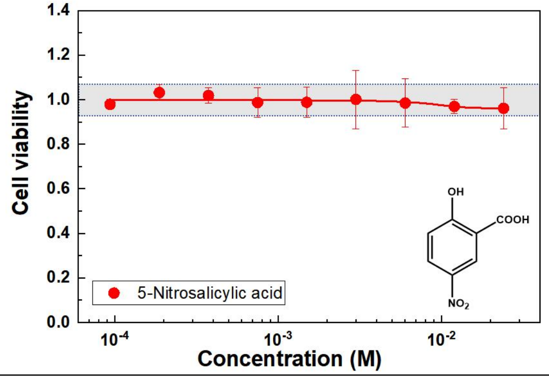	ND	0
**Phenols (n = 4)**			
Hydroquinone	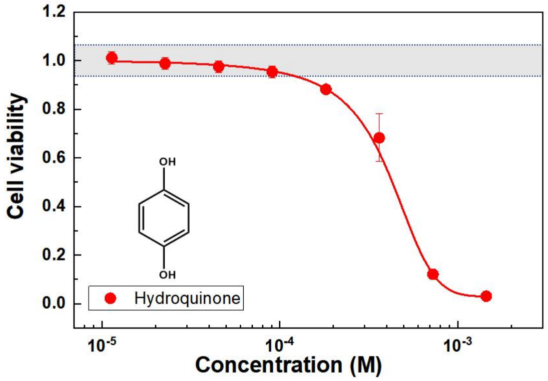	$(4.30 \pm 0.01) \times 10^{-4}$	0
Phenol	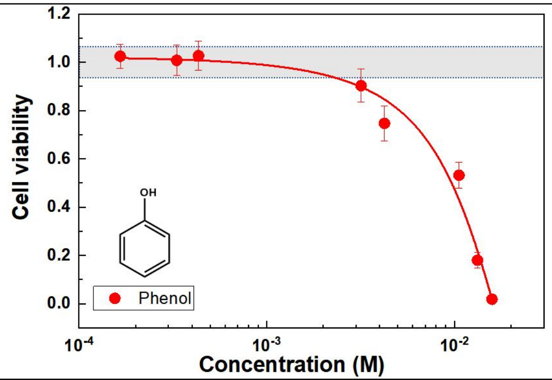	$(9.67 \pm 0.49) \times 10^{-4}$	0

2-Phenylphenol	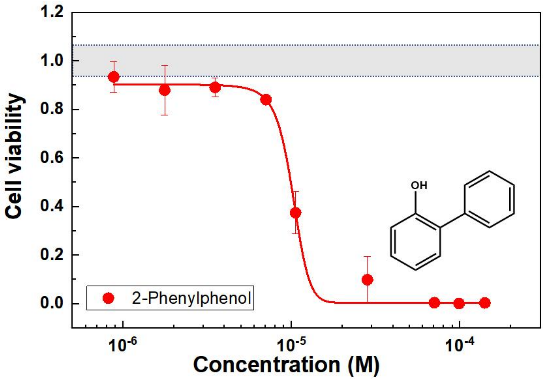	$(9.90 \pm 0.45) \times 10^{-6}$	1
**Oxo-Polycyclic Aromatic Hydrocarbons (OPAHs) (n = 3)**			
Acenaphthenequinone	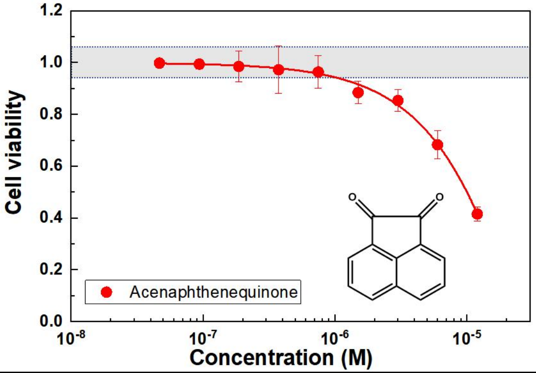	$(9.99 \pm 0.46) \times 10^{-6}$	1
9-Fluorenone	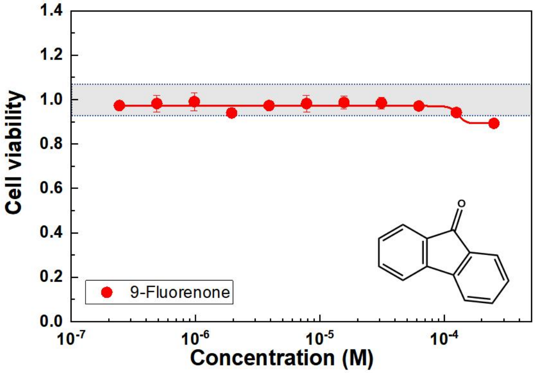	ND	0

2-Hydroxy-9,10-anthracen-edione	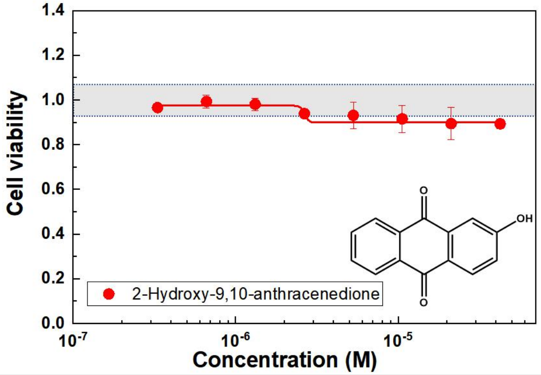	ND	0

[a] ND: Not detected. IC_{50} could not be determined within the tested concentration range.

References

Damiani, E., Solorio, J. A., Doyle, A. P., Wallace, H. M. How reliable are *in vitro* IC_{50} values? Values with cytotoxicity assay in human glioblastoma cells. *Toxicol. Lett*, **2019**, *302*, 28-34.

Fuller, S. J., Zhao, Y., Cliff, S. S., Wexler, A. S., Kalberer, M. Direct surface analysis of time-resolved aerosol impactor samples with ultrahigh-resolution mass spectrometry, *Anal. Chem.*, **2012**, *84(22)*, 9858-9864.

Kourtchev, I., Fuller, S., Aalto, J., Ruuskanen, T. M., Mcleod, M. W., Maenhaut, W., Jones, R., Kulmala, M., Kalberer, M. Molecular composition of boreal forest aerosol from Hyytiala, Finland, using ultrahigh resolution mass spectrometry. *Environ. Sci. Technol.*, **2013**, *47(9)*, 4069-4079.

Peets, P., Wang, W.-C., MacLeod, M., Breitholtz, M., Martin, J.W., Kruve, A. MS2Tox machine learning tool for predicting the ecotoxicity of unidentified chemicals in water by nontarget LC-HRMS. *Environ. Sci. Technol.*, **2022**, *56*, 15508-15517.

Ruttkies, C.; Schymanski, E. L.; Wolf, S.; Hollender, J.; Neumann, S. MetFrag relaunched: incorporating strategies beyond *in silico* fragmentation. *J. Cheminf.*, **2016**, *8(1)*, 3.

Schymanski, E. L.; Jeon, J.; Gulde, R.; Fenner, K.; Ruff, M.; Singer, H. P.; Hollender, J. Identifying small molecules via high resolution mass spectrometry: communicating confidence. *Environ. Sci. Technol.*, **2014**, 48(4), 2097-2098.

Wang, K., Zhang, Y., Huang, R. J., Wang, M., Ni, H., Kampf, C. J., Cheng, Y., Blide,

M., Glasius, M., Hoffmann, T. Molecular characterization and source identification of atmospheric particulate organosulfates using ultrahigh resolution mass spectrometry. *Environ. Sci. Technol.*, **2019**, *53(11)*, 6192-6202.

Zou, Y. J., Jin, C. Y., Su, Y., Li, J., Zhu, B. S. Water soluble and insoluble components of urban $PM_{2.5}$ and their cytotoxic effects on epithelial cells (A549) *in vitro*. *Environ. Pollut.*, **2016**, *212*, 627-635.
